# High-Flow Humidified Oxygen as an Early Intervention in Children With Acute Severe Asthma: Protocol for a Feasibility Randomized Controlled Trial

**DOI:** 10.2196/54081

**Published:** 2024-03-28

**Authors:** Hector Rojas-Anaya, Akshat Kapur, Graham Roberts, Damian Roland, Atul Gupta, Michaela Lazner, Jane Bayreuther, John Pappachan, Christina Jones, Stephen Bremner, Fleur Cantle, Paul Seddon

**Affiliations:** 1 University Hospitals Sussex National Health Service Foundation Trust Brighton United Kingdom; 2 Brighton and Sussex Clinical Trials Unit Brighton and Sussex Medical School Brighton United Kingdom; 3 Respiratory Care Royal Alexandra Children's Hospital University Hospitals Sussex National Health Service Foundation Trust Brighton United Kingdom; 4 Department of Paediatric Allergy and Respiratory Medicine University of Southampton Southampton United Kingdom; 5 National Institute for Heath Research Southampton Biomedical Research Centre University Hospital Southampton National Health Service Foundation Trust Southampton United Kingdom; 6 Paediatric Emergency Medicine Leicester Academic Group Children’s Emergency Department Leicester Royal Infirmary Leicester United Kingdom; 7 Social science APPlied Healthcare and Improvement REsearch Group Department of Population Health Sciences Leicester University Leicester United Kingdom; 8 Paediatric Respiratory Medicine King's College Hospital London United Kingdom; 9 Children's Emergency Department Royal Alexandra Children's Hospital University Hospitals Sussex National Health Service Foundation Trust Brighton United Kingdom; 10 Children's Emergency Department University Hospital Southampton National Health Service Foundation Trust Southampton United Kingdom; 11 School of Psychology Faculty of Health and Medical Sciences University of Surrey Guildford United Kingdom; 12 Emergency Department King's College Hospital London United Kingdom

**Keywords:** asthma, child, wheezing, oxygen therapy, high-flow humidified oxygen therapy

## Abstract

**Background:**

Acute severe asthma (ASA) is a leading cause of hospital attendance in children. Standard first-line therapy consists of high-dose inhaled bronchodilators plus oral corticosteroids. Treatment for children who fail to respond to first-line therapy is problematic: the use of intravenous agents is inconsistent, and side effects are frequent. High-flow humidified oxygen (HiFlo) is widely used in respiratory conditions and is increasingly being used in ASA, but with little evidence for its effectiveness. A well-designed, adequately powered randomized controlled trial (RCT) of HiFlo therapy in ASA is urgently needed, and feasibility data are required to plan such an RCT. In this study, we describe the protocol for a feasibility study designed to fill this knowledge gap.

**Objective:**

This study aims to establish whether a full RCT of early HiFlo therapy in children with ASA can be conducted successfully and safely, to establish whether recruitment using deferred consent is practicable, and to define appropriate outcome measures and sample sizes for a definitive RCT. The underlying hypothesis is that early HiFlo therapy in ASA will reduce the need for more invasive treatments, allow faster recovery and discharge from hospital, and in both these ways reduce distress to children and their families.

**Methods:**

We conducted a feasibility RCT with deferred consent to assess the use of early HiFlo therapy in children aged 2 to 11 years with acute severe wheeze not responding to burst therapy (ie, high-dose inhaled salbutamol with or without ipratropium). Children with a Preschool Respiratory Assessment Measure score ≥5 after burst therapy were randomized to commence HiFlo therapy or follow standard care. The candidate primary outcomes assessed were treatment failure requiring escalation and time to meet hospital discharge criteria. Patient and parent experiences were also assessed using questionnaires and telephone interviews.

**Results:**

The trial was opened to recruitment in February 2020 but was paused for 15 months owing to the COVID-19 pandemic. The trial was reopened at the lead site in July 2021 and opened at the other 3 sites from August to December 2022. Recruitment was completed in June 2023.

**Conclusions:**

This feasibility RCT of early HiFlo therapy in children with ASA recruited to the target despite major disturbances owing to the COVID-19 pandemic. The data are currently being analyzed and will be published separately.

**Trial Registration:**

International Standard Randomised Controlled Trial Number Registry ISRCTN78297040; https://www.isrctn.com/ISRCTN78297040

**International Registered Report Identifier (IRRID):**

DERR1-10.2196/54081

## Introduction

### Background

Asthma is a common chronic disorder of reversible airway obstruction characterized by bronchial smooth muscle contraction, airway inflammation, and increased airway secretion [1]. It is the most common noncommunicable disease in childhood, affecting 1 in 10 children worldwide [2] and causing >1000 deaths per day [3]. Children with asthma are prone to episodes of acute severe airway obstruction, characterized by wheezing and increased work of breathing, and often require hospital treatment. Acute severe asthma (ASA) is a leading cause of hospital attendance in children, accounting for up to 7% of all pediatric emergency visits [4] and 8.5% of pediatric admissions from emergency departments (EDs) [5], the most common single cause. Many preschool children who have not yet been diagnosed with asthma are admitted to the hospital with episodes of acute severe wheezing. They present identically and are treated in the same way as older children diagnosed with asthma, although they can be less responsive to therapy [6]. In this paper, the term ASA is used to describe children presenting with acute wheezing and breathing difficulty, whether they have an established diagnosis of asthma.

Therapy for ASA is directed at (1) relieving bronchoconstriction with bronchodilators, (2) decreasing airway inflammation with corticosteroids, and (3) clearing airway secretions so that they do not become thick and block the airways. Standard first-line emergency treatment [7] for ASA in children starts with burst therapy in the first hour (3 doses of high-dose inhaled salbutamol, sometimes with inhaled ipratropium, via a spacer device or nebulizer) and oral corticosteroids. During the next 1 to 4 hours, many children improve clinically and may be discharged. However, some children fail to respond to standard therapy and require hospital admission for more intensive, second-line treatment; without effective treatment, these children are at risk of fatigue, respiratory failure, and death [8]. Second-line treatment commonly includes intravenous bronchodilators (≥1 of aminophylline, salbutamol, and magnesium sulfate). However, evidence for the efficacy of such treatments is limited and inconsistent, with frequent side effects, including tachycardia, jitteriness, tremor, palpitations, nausea, vomiting, elevated lactate level, and hypokalemia [7], which can cause considerable distress to the child and family. Current guidelines [7,9] provide little guidance (because of the scarcity of evidence) regarding which second-line treatment clinicians should use. Therefore, there is a need to investigate other options for treating ASA to improve the effectiveness of the treatment and reduce adverse effects.

High-flow humidified oxygen (HiFlo) therapy is an innovative health care technology that supports breathing by supplying a warm, humidified mixture of air and oxygen at high-flow rates via fine nasal cannulae that has shown promising results in other acute respiratory conditions in children [10]. Traditional oxygen therapy uses cold, unhumidified oxygen directly from a cylinder or a wall outlet. Although this is helpful in improving oxygenation, it is uncomfortable for patients and causes drying and cooling of the nose and mouth, and potentially of the lower airways, which can cause worsening of airway obstruction and even airway damage. Therefore, unmodified oxygen therapy can only be delivered at very low flow rates. Using HiFlo technology, the air or oxygen percentage mix can be varied; it is warmed to body temperature and delivered at 100% humidity. As a result, much higher flows can be delivered without discomfort or adverse effects on the airways.

There is now considerable experience with the use of this technology in both adults and children [10]. Most of the clinical experience and evidence for the efficacy of HiFlo therapy in children is derived from studies performed in preterm neonates with surfactant deficiency. In this population, HiFlo therapy appears to be as effective as continuous positive airway pressure (CPAP) therapy and has become a standard therapy [11]. The physiological basis of its effectiveness is unclear [10,12]: HiFlo therapy itself may generate CPAP [13,14], but it may also reduce nasopharyngeal dead space, reduce upper airway resistance, and reduce the metabolic demand required to humidify inspired gases [15]. In recent years, there has been increased use of HiFlo therapy in infants with acute bronchiolitis [16]. Retrospective studies have suggested that introducing HiFlo therapy for acute bronchiolitis is associated with a reduced need for intubation [17,18]. Prospective trials comparing HiFlo with standard bronchiolitis therapy (low-flow 100% oxygen) have shown improved oxygen saturation levels [19], fewer treatment failures [20,21], and a nonsignificant trend toward faster weaning from oxygen [20]. A Cochrane systematic review concluded that HiFlo therapy is feasible and well-tolerated in infants with bronchiolitis but that further evidence for its effectiveness is needed [22].

To date, there have been no substantial randomized controlled trials (RCTs) of HiFlo therapy in children with ASA, although its use in ASA has been rapidly increasing [23]. The pathophysiology of ASA is very different from that of bronchiolitis. Bronchiolitis is characterized by more mechanical distal airway obstruction [22], whereas in ASA, bronchial smooth muscle constriction plays a major role [1,6]. Retrospective observational studies of HiFlo therapy in children with ASA have suggested improvements in physiological parameters and asthma severity scores [24,25] but have also raised concerns that using HiFlo therapy may delay the initiation of other forms of respiratory support [26]. There have been 2 small single-center pilot RCTs on HiFlo therapy in children with acute asthma. Ballestero et al [27] randomized 62 children with ASA with acute respiratory failure to HiFlo therapy versus conventional oxygen therapy. The asthma score of a higher proportion of children on HiFlo therapy reduced (*pulmonary score*—unreferenced) by 2 points in the first 2 hours of treatment; however, there was no difference in the need for admission or length of hospital stay. Gauto Benítez et al [28] randomized 65 children in a single center in Paraguay to HiFlo therapy or conventional oxygen therapy: the inclusion criteria were somewhat unclear, and both groups received continuous intravenous magnesium in addition to inhaled bronchodilators as a standard practice in this institution. They found no difference in the proportion reducing their asthma score (*pulmonary index score*—again unreferenced) by 2 points or in the length of hospital stay. A recent review of HiFlo in children with ASA by Chao et al [23] concluded that “large well-designed randomized controlled trials assessing the clinical efficacy of high-flow nasal cannula oxygen for children with asthma exacerbation are urgently warranted.” The evidence from published studies is encouraging but mixed and is lacking in clinical outcomes. It does not provide the feasibility information required to plan an RCT on the clinical effectiveness of early HiFlo therapy in childhood ASA.

In summary, ASA in childhood is a common emergency condition with important impacts on health care costs and quality of life and presents a risk to life. HiFlo is a novel therapy that has the potential to treat patients with ASA more effectively and reduce hospital stays and intensive care admissions. However, its use is becoming widespread in clinical practice despite a lack of good evidence. If HiFlo therapy in patients with ASA is not evaluated objectively, there is a risk that a treatment without proven benefits (but with significant costs) may drift into widespread practice. Therefore, there is an urgent need for a well-designed, adequately powered RCT of HiFlo therapy in patients with ASA. To plan such an RCT, feasibility data are required. A definitive RCT would be large and expensive to run, and it is unclear whether it would be feasible, how large it would need to be, and what are the most appropriate outcome measures. This paper presents the protocol of a feasibility study designed to fill this knowledge gap, which has been successfully executed in 4 children’s hospitals in the United Kingdom. The formal results will be published separately.

### Study Aim and Feasibility Objectives

This feasibility study aimed to establish whether a full RCT of early HiFlo therapy in children with ASA can be conducted successfully and safely and whether recruitment to such a trial, using deferred consent, is practicable in children aged 2 to 11 years presenting to the hospital with ASA. The underlying hypothesis is that early HiFlo therapy in children with ASA will reduce the need for more invasive treatments, allow faster recovery and discharge from hospital and, in both these ways, reduce distress to children and their families. The trial was designed to generate the data required to plan a definitive RCT that would satisfy the clinical and health economic end points and the requirements of children, parents, clinicians, and health services.

## Methods

### Primary Feasibility Objectives and Outcome Measures

A total of 6 feasibility objectives and associated outcome measures (Table 1) were established to help determine the feasibility of progressing to a full RCT, which would require the following 4 conditions to be met:

1. At least 50% enrollment rate among eligible children (feasibility outcome 1)
2. At least 70% deferred consent rate [29] (feasibility outcome 2)
3. At least 80% of data collection is complete per participant for candidate primary outcome measures (feasibility outcome 3)
4. Confirmation that the predicted sample size, number of centers, and recruitment rates would allow an appropriately powered RCT to be conducted in the United Kingdom for 3 years (feasibility outcome 5)

Discussions with colleagues indicated that at least 15 large UK pediatric centers would be interested in participating in a definitive RCT on this question. The study has been discussed with two relevant research networks: (1) the UK National Institute for Health and Care Research (NIHR) Children Respiratory and Cystic Fibrosis Clinical Studies Group and (2) Pediatric Emergency Research in the United Kingdom and Ireland [30], a network of research-active pediatric emergency care clinicians who have indicated that they will facilitate the process of identifying appropriate centers for the definitive study.

The 2 candidate primary outcome measures recorded and evaluated are provided in Textbox 1.

**Table 1:** Primary feasibility objectives and outcome measures.

Feasibility objectives	Feasibility outcome measures	Time point of evaluation
To evaluate enrollment rates	Proportion of enrolled (ie, randomized) children among eligible patients with ASA^a^	Enrollment
To evaluate deferred consent rates	Proportion of children with signed deferred consent among those enrolled into the study	Deferred consent
To assess feasibility of recording candidate primary outcome measures	Proportion of data collection completed per participant for the 2 candidate primary outcome measures	Discharge
To estimate the variability of candidate primary outcome measures	Summary statistics for the 2 candidate primary outcome measures	Discharge
To determine design characteristics for a subsequent definitive study	Proposed design, sample size, and number of centers for a definitive study	End of study
To assess the acceptability of HiFlo^b^ therapy and the deferred consent model to children, parents, and staff	Satisfaction ratings on the end-of-study questionnaire	Discharge

^a^ASA: acute severe asthma.

^b^HiFlo: high-flow humidified oxygen.

**Candidate primary outcome measures.**

- Treatment failure needing escalation of therapy as defined in the *Treatment Failure and Escalation* section.
- The time (h) between presentation to the emergency department and meeting hospital discharge criteria as defined by the following criteria:
  The ability of the child to maintain arterial oxygen saturation (SpO_2_) measured by pulse oximeter at ≥92% without supplemental oxygen or respiratory support for a 4-hour period
  The ability of the child to remain clinically stable for a minimum of 4 hours between inhaled bronchodilator doses
  The ability to maintain these conditions continuously until hospital discharge

### Treatment Failure and Escalation

Textbox 2 lists the criteria recorded for treatment failure needing escalation in therapy.

Escalations in therapy for the primary analysis are described in Textbox 3. Pragmatically, senior clinicians on duty managing these patients had the discretion to escalate treatment if deemed clinically appropriate and justified but were asked to clearly state the reason for escalation using the abovementioned criteria. The order in which escalations are listed in Textbox 3 does not imply that they needed to be implemented in that order. The clinicians were free, for example, to implement HiFlo therapy in the standard care group before administering the second or third intravenous agent.

For the primary analysis, administering a first intravenous agent is not categorized as *treatment failure requiring escalation* in the standard care group because starting an intravenous agent would be the standard following step in a child who fails burst therapy. However, it was possible that some children randomized to the standard care group may not have received an intravenous agent directly.

Furthermore, it was useful to examine whether commencing early HiFlo therapy has an effect on the total burden of invasive treatments required. Therefore, a secondary analysis, in which escalations of therapy are defined as in Textbox 4, was carried out.

The candidate secondary outcome measures are provided in Textbox 5.

**Criteria for treatment failure needing escalation in therapy.**

- Preschool Respiratory Assessment Measure (PRAM) score [31] rising or not falling
- Respiratory rate rising or not falling
- Heart rate rising or not falling
- Rising oxygen requirement
- Rising partial pressure of carbon dioxide (pCO_2_) in capillary, venous, or arterial blood
- Other clinical concerns (specified)

**Escalations in therapy for primary analysis.**

**High-flow humidified oxygen (HiFlo) group**

- Commencing intravenous bronchodilator therapy
- Commencing the second or third intravenous agent
- Re-escalating inhaled bronchodilator therapy to an hourly or more frequent dosage
- Commencing noninvasive ventilation with bilevel positive airway pressure (BiPAP) ventilation
- Intubation for invasive ventilation

**Standard care group**

- Commencing HiFlo therapy
- Commencing the second or third intravenous agent
- Re-escalating inhaled bronchodilator therapy to an hourly or more frequent dosage
- Commencing noninvasive ventilation with BiPAP ventilation
- Intubation for invasive ventilation

**Escalations in therapy for secondary analysis.**

**High-flow humidified oxygen (HiFlo) group**

- Commencing administration of the first, second, or third intravenous agent
- Re-escalating inhaled bronchodilator therapy to an hourly or more frequent dosage
- Commencing noninvasive ventilation with bilevel positive airway pressure (BiPAP) ventilation
- Intubation for invasive ventilation

**Standard care group**

- Commencing administration of the first, second, or third intravenous agent
- Commencing HiFlo therapy
- Re-escalating inhaled bronchodilator therapy to an hourly or more frequent dosage
- Commencing noninvasive ventilation with BiPAP ventilation
- Intubation for invasive ventilation

**Candidate secondary outcome measures.**

- Time (h) between presentation to the emergency department (ED) and the actual hospital discharge
- Time (h) between presentation to the ED and achieving a Preschool Respiratory Assessment Measure (PRAM) [31] score of ≤3
- Time (h) between presentation to the ED and the ability to maintain oxygen saturation (SpO_2_) at ≥92% without supplemental oxygen or respiratory support
- Need for intravenous bronchodilator therapy
- Duration of intravenous bronchodilator therapy
- Requirement for noninvasive ventilation
- Requirement for invasive ventilation (intubation)
- Treatment-related adverse effects:
  Intravenous or inhaled bronchodilator–related side effects (eg, vomiting, tachycardia, and lactic acidosis)
  Poor compliance with HiFlo therapy
- Hospital readmission within 48 hours of discharge
- Acceptability and comfort scores for treatment during the episode (recorded using the end-of-study questionnaire and qualitative interview following the episode). These measures were codeveloped with the Lived Experience Advisory Panel before the trial commenced.

### Study Design and Setting

This was a multicenter feasibility RCT of 50 children in the following 4 National Health Service (NHS) Trusts in England:

- The University Hospitals Sussex (UHSx) NHS Foundation Trust, Royal Alexandra Children’s Hospital, Brighton
- The King’s College Hospital NHS Foundation Trust, London
- The University Hospital Southampton NHS Foundation Trust, Southampton
- The University Hospitals of Leicester NHS Trust, Leicester Royal Infirmary, Leicester

**Figure 1:**
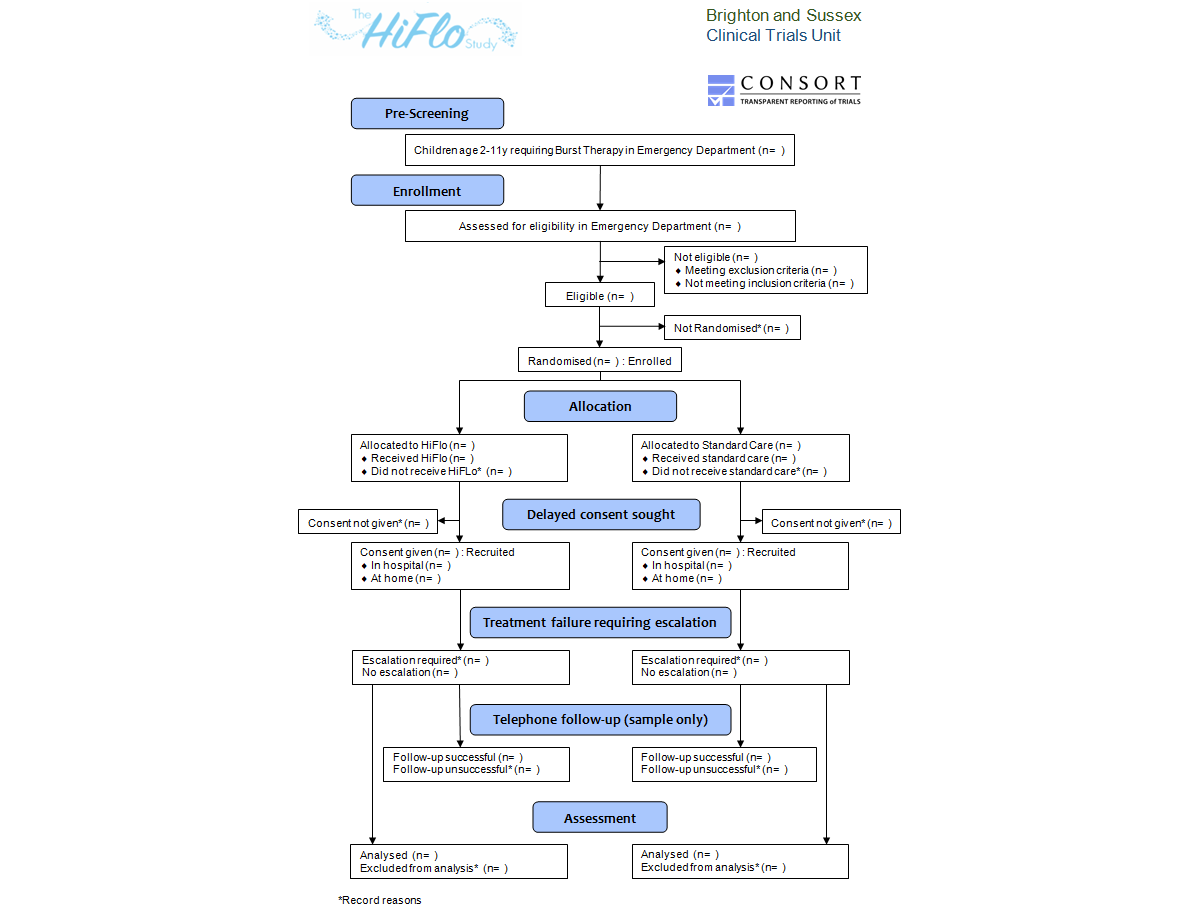
CONSORT (Consolidated Standards of Reporting Trials) diagram of the study. HiFlo: high-flow humidified oxygen.

Eligible children were randomized to the intervention (HiFlo therapy) or control (standard care) arms, as shown in the CONSORT (Consolidated Standards of Reporting Trials) diagram (Figure 1). Screening, recruitment, and randomization were performed in the relevant EDs of the participating hospitals. Randomization was stratified by site, age (<5 years and ≥5 years), and severity of acute asthma (the Preschool Respiratory Assessment Measure [PRAM] score at study entry: <8 and ≥8, refer to the *PRAM Scoring* section), with an equal ratio between both arms.

The study was pragmatic, with HiFlo therapy being an add-on to the existing therapy in those randomized to the intervention arm, and was clearly not blinded as this would have been impossible. The children were not denied access to the existing standard second-line interventions (eg, intravenous bronchodilators) because they participated in the study. The treating clinical team was allowed to initiate intravenous bronchodilators as clinically indicated in either treatment arm. In children randomized to the intervention arm, HiFlo therapy was commenced as soon as possible after randomization as the subsequent treatment was initiated rather than intravenous bronchodilators. As the existing treatment guidelines [7,9] make no specific recommendations and because the choice of intravenous bronchodilators is physician dependent across the 4 institutions, the study protocol was physician led and did not specify which intravenous bronchodilator is initiated first. Similarly, if a child randomized to the standard care arm was failing to respond, as defined by the preset criteria, the clinical team could opt to initiate HiFlo as rescue therapy—the child remained in the study on an intention-to-treat basis. The reasons for discontinuing the intervention prematurely or for other protocol violations were recorded.

### Sample Size and Planned Recruitment Rate

The size of the study was determined by the number of children required to provide an accurate estimate of the variability in the candidate primary outcome measures: recommendations for this vary between 50 [32] and 70 [33]. The larger number was chosen to allow 30% attrition to deferred consent [29]. Therefore, the study originally aimed to recruit 70 children aged <18 months. The recruitment target was subsequently lowered to 48 after an agreement with the NIHR Clinical Research Network that this number was sufficient to meet the objectives of the feasibility RCT. Initially, the recruitment was planned to be from 3 collaborating centers; the fourth site (Leicester) was subsequently added. The subsequent definitive RCT will determine whether HiFlo therapy is an effective intervention for children with ASA.

### Participants and Eligibility Criteria

Children aged 2 to 11 years were eligible if they presented to the ED with ASA and failed to respond to standard first-line therapy (high-dose inhaled bronchodilators) based on the eligibility criteria listed in Textbox 6.

The PRAM was chosen because it has been validated across the age range of intended participants [31] and has been shown to be a good predictor of the need for admission and escalation of therapy [35]. A child with a PRAM score of 5 would typically have moderately increased work of breathing, audible wheeze, and oxygen saturation <92% but >90%. The threshold of PRAM score ≥5 was selected based on the advice of the team that designed PRAM (Professor Francine Ducharme, MD, personal communication, April 2019) and because this threshold had been successfully used as an entry criterion in a previous trial of administering nebulized magnesium to patients with acute asthma [36].

**Inclusion and exclusion criteria.**

**Inclusion criteria**

- Participants having an acceptable individual capable of giving consent on their behalf (eg, parent or guardian of a child aged <16 years)
- Participants aged 2 to 11 years
- ASA, defined as respiratory distress combined with wheezing on auscultation (a formal preceding diagnosis of asthma was not necessary)
- Failure to respond to standard initial emergency management [7] with burst therapy (back-to-back 3 consecutive inhaled or nebulized doses of salbutamol with or without the addition of ipratropium bromide for a 1-h period) and systemic corticosteroids, with or without subsequent intravenous bronchodilator therapy as deemed appropriate by the treating physician. Failure to respond will be defined as a Preschool Respiratory Assessment Measure (PRAM) score of ≥5 between 1 hour and 4 hours after starting burst therapy.

**Exclusion criteria**

- Clinical or radiological evidence of bacterial pneumonia: fever >38.5°C and focal signs on auscultation or chest x-ray
- Signs of impending respiratory failure mandating immediate intubation. These were at the discretion of the treating clinical team but included elevated pCO_2_, refractory hypoxemia, and exhaustion
- Contraindications to the use of HiFlo therapy are as follows:
  Air leak (eg, pneumothorax, pneumomediastinum, or subcutaneous emphysema)
  Decreased level of consciousness—AVPU (Alert, Voice, Pain, Unresponsive) score [34] P or worse
  Recent (within 6 wk) bowel surgery
  Intractable vomiting
- Other major respiratory, cardiovascular, or neurological condition
- Previous participation in the HiFlo ASA study during a prior hospital episode

### Study Procedures

This section summarizes the procedures and evaluations conducted to support the feasibility objectives. The timing of the procedures in relation to the established study visits is given in Table 2. A detailed description of each procedure is provided in Section 6 of the study protocol in Multimedia Appendix 1 [1,4-8,10,11,13-15,17-24,27,29,31-33,37-49].

**Table 2:** Schedule of events.

Study procedures	Visit				
	Screening	Enrollment	Treatment	Hospital discharge	Follow-up
Inform parents regarding the study (eg, poster and leaflet)	✓		✓		
Eligibility assessment	✓				
Demographics and medical history	✓				
Observations, including physical examination, vital signs, PRAM^a^ scoring, and oxygen requirement	✓		✓		
Eligibility check and randomization		✓			
Early HiFlo^b^ or standard therapy (including treatment escalation and weaning)			✓		
Deferred informed consent			✓		
Routine blood investigations			✓		
Concomitant medications			✓		
Adverse event assessments			✓		
CRF^c^ completion and data query resolution			✓	✓	✓
End-of-study questionnaire				✓	
Qualitative interviews with health care professionals and parents					✓

^a^PRAM: Preschool Respiratory Assessment Measure.

^b^HiFlo: high-flow humidified oxygen.

^c^CRF: Case Report Form.

### Screening

All children arriving at the participating EDs were routinely triaged by an experienced nurse and reviewed clinically during and after completing burst therapy. Children potentially eligible for the study were identified and actively screened for inclusion by ED clinical staff or research nurses according to their local capacity. Screening information included Trust ID number, age, and reasons for not being eligible for trial participation or if they were eligible but declined participation.

### Enrollment and Randomization

The children who met all inclusion criteria and had no exclusion criteria were enrolled in the study and randomized to the intervention (early HiFlo therapy) or control (conventional therapy) arms only after the confirmation of eligibility by a treating clinician delegated to conduct this task. Randomization occurred at enrollment and before consent, as explained in the *Deferred Consent and Recruitment* section; it was implemented using the *Sealed Envelope* (Sealed Envelope Ltd) web-based randomization software [50] and conducted by a member of the research team trained in the study.

### Treatment

The intervention was an add-on to standard care. The key difference between the groups in the 2 arms was the early use of HiFlo therapy, that is, starting HiFlo therapy as the next measure after the failure of burst therapy, and it is this strategy which was examined.

### HiFlo Intervention

**Figure 2:**
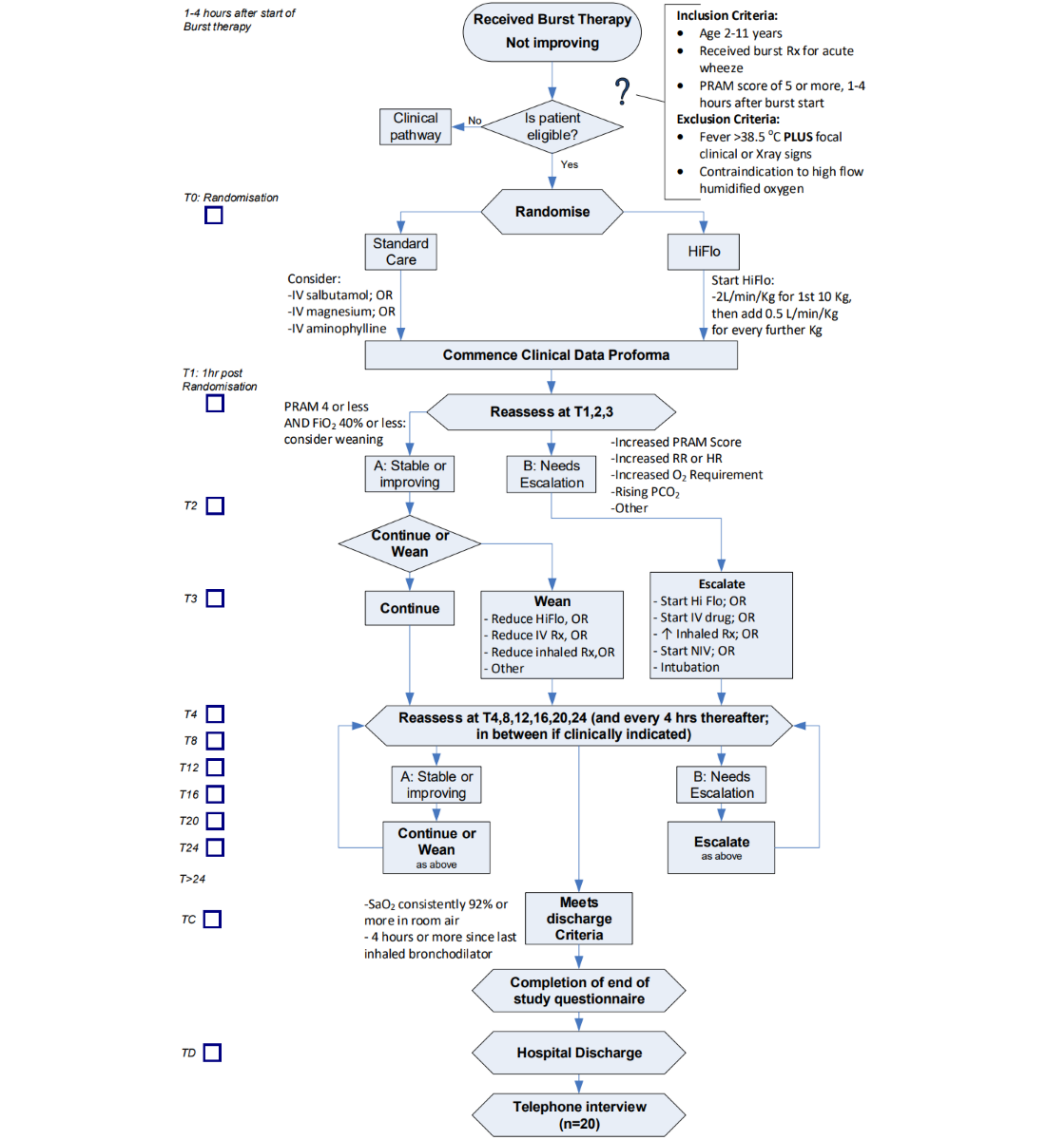
Flowchart of the study. FiO_2_: fraction of inspired oxygen; HiFlo: high-flow humidified oxygen; HR: heart rate; IV: intravenous; NIV: noninvasive ventilation; O_2_: oxygen; pCO_2_: partial pressure of carbon dioxide; PRAM: Preschool Respiratory Assessment Measure; RR: respiratory rate; SaO_2_: arterial oxygen saturation.

The use of HiFlo therapy in the study followed established practices and was standardized as far as possible while allowing for clinical judgment (Figure 2). The reasons for escalating and reducing treatment were recorded, and these data will help in defining treatment escalation and weaning pathways for the definitive RCT. The HiFlo therapy was commenced at a flow rate of 2 L/kg/min for the first 10 kg of body weight, with an additional flow rate of 0.5 L/kg/min for every kilogram of body weight >10 kg to a maximum absolute flow rate of 40 L/min, and fraction of inspired oxygen (FiO_2_) was adjusted appropriately to maintain oxygen saturation ≥92%. At the discretion of the treating clinician, the flow could be increased to 3 L/kg/min but again with a maximum absolute flow rate of 40 L/min.

### Equipment

Vapotherm, a manufacturer of portable and fixed HiFlo equipment, provided sufficient Precision Flow Plus units and consumables to make the early HiFlo intervention available free of cost in the 4 participating hospitals for the duration of the study.

Aerogen provided in-line Aerogen solo nebulizers and controller units to be used with all Vapotherm systems during the study.

### HiFlo Therapy Training

HiFlo therapy was already in use in high-dependency units (HDUs, EDs) in all participating hospitals, and further staff training was undertaken in the setup period before the start of the study. HiFlo hands-on sessions were organized for staff from the EDs, HDUs, and relevant or equivalent clinical wards of participating centers by the UK Vapotherm representative Solus Medical Limited, with the aim of having at least 1 member of staff fully trained per shift. Local nursing and clinical staff members were trained as *super users* to support with opportunistic training, retraining, and on-site competency checks.

### Weaning From HiFlo

Weaning commenced once the child was clinically stable, according to the standard criteria agreed upon across the 4 centers. The weaning strategy was not rigidly protocolized but broadly followed the schema published in the protocol for the FIRST-ABC (FIRST-line support for assistance in breathing in children) study by Richards-Belle et al [51]. Essentially, the reduction in FiO_2_ occurred first, and then the flow rate was reduced in a stepwise manner once FiO_2_ was consistently ≤40% (Figure 2).

### Deferred Consent and Recruitment

A deferred consent model was used to avoid delay in treatment and minimize distress to families who presented to the ED with acutely unwell children. The consent process is summarized in this section (complete details are provided in section 6.4 of the study protocol in Multimedia Appendix 1).

Informed consent was not sought before randomization, but the parents were approached for informed consent within a maximum of 72 hours of randomization once their child’s condition was stable. The parents were approached for informed consent by trained staff from the direct care team, who explained the study and provided information sheets to the parents and children (if age appropriate). The parents were given time to read and understand the information and the opportunity to clarify any questions regarding the study and their child’s participation. Written informed consent was obtained from all the parents.

If a child was discharged from hospital before parents could be approached for deferred consent, they were contacted by a trained research nurse who explained the study via phone. Written participant information and consent forms were then sent out by post. This model was successfully used, for example, in the Emergency treatment with levetiracetam or phenytoin in status epilepticus in children (EcLIPSE) study by Lyttle et al [52], and feedback from parents was positive [37]. There was a possibility for parents to provide verbal consent via phone in special circumstances. A special process for the sensitive case of having to approach bereaved parents for consent was designed.

If neither written nor verbal consent was received, the child was not recruited into the study, and the data collected were not included in the study analysis.

### Hospital Discharge

#### Hospital Discharge Criteria

It was recognized that the timing of discharge from hospital is affected by multiple factors in addition to the child’s medical condition; therefore, criteria of fitness for discharge were defined as a more robust and reproducible candidate primary outcome measure in addition to actual hospital discharge, which was used as a candidate secondary outcome measure. Hospital discharge criteria are defined in Textbox 7.

**Hospital discharge criteria.**

- The ability of the child to maintain arterial oxygen saturation (SpO_2_) measured by pulse oximeter at ≥92% without supplemental oxygen or respiratory support for a 4-hour period
- The ability of the child to remain clinically stable for a minimum of 4 hours between inhaled bronchodilator doses
- Maintaining these conditions continuously until hospital discharge

#### End-of-Study Questionnaire

The study included a patient satisfaction questionnaire, tailored to children with acute severe wheezing, for all parents and their children to be collected at the time of hospital discharge.

Although validated measures of satisfaction existed within the ED setting, these were not tailored to the case of children with acute severe wheezing. Therefore, questionnaire items measuring global satisfaction outcomes were adapted with the help of patient and public involvement (PPI) groups (refer to the *PPI group* section).

The items related to treatment effectiveness, treatment satisfaction, service satisfaction, information and consent, physical comfort, pain, and communication [38] were included in the questionnaire to be rated on a Likert or visual analog scale for parents and using pictographic tools, similar to the FACES pain scale [39] and the widely used childhood Asthma-Control Test [40] for children aged ≥4 years.

#### Follow-Up (Telephone Interviews)

A qualitative substudy was incorporated to explore the acceptability of HiFlo therapy compared with conventional therapy and the acceptability of the deferred consent process among parents and health care professionals. Parents and health care professionals across the 4 sites were invited to participate in a semistructured telephone interview with an experienced qualitative researcher to elicit their views and opinions of the therapy and the study more generally. All telephone interviews lasted for a maximum of 30 minutes and were recorded for later transcribing. Section 6.7 of the study protocol in Multimedia Appendix 1 presents a detailed account of the recruitment and interview process and the qualitative analysis followed during this substudy. A topic guide for the interviews presented in Textbox 8 was devised with the assistance of the Lived Experience Advisory Panel (LEAP) described in the *PPI group* section.

**Topic guide for qualitative interviews with parents and health care professionals (HCPs).**

1. How acceptable did parents, children, and health professionals find the treatment approach used in this study?
2. What aspects of the treatment and the study more generally worked well?
3. What aspects of the treatment and the study more generally needed improvement?
4. If applicable, how did the treatment approach differ from those experienced in the past?
5. What would parents, children, and HCPs change about the therapy or study more generally?
6. Were there any outcomes which weren’t measured which should have been?
7. What did parents, children, and HCPs think about the deferred consent process?
8. What would encourage other parents, children, and HCPs to participate in this study?

#### Clinical Data Recording

The standard of care was guided by a well-defined wheezing or asthma care pathway for children agreed upon by the participating centers, which included various observations to aid with treatment decision-making. Key observations and assessments conducted and recorded for this study are summarized in Textbox 9 (a detailed description is available in section 6.7 of the study protocol in Multimedia Appendix 1).

**Trial observations and assessments.**

- Physical examination: this includes evaluation of suprasternal retraction, scalene muscle contraction, air entry, wheezing, work of breathing (respiratory distress), chest findings, and cardiovascular system findings.
- Vital signs: vital signs at initial assessment (triage) and during subsequent reassessments include respiratory rate, heart rate, oxygen saturation (SpO_2_), capillary refill time, and temperature.
- Preschool Respiratory Assessment Measure scoring: assessments required involve a physical examination and pulse oximetry, which are all routine procedures in the participating centers.
- Oxygen requirement: this includes monitoring of oxygen (O_2_) flow and fraction of inspired oxygen (FiO_2_).
- Blood gases: a proportion of patients may have blood gas measurements performed routinely. Specifically, partial pressure of carbon dioxide (pCO_2_) results (if available) will be used for treatment escalation decisions. In children, these will normally be measured using capillary or venous blood.

#### PRAM Scoring and Training

Progress was monitored regularly from hospital admission until discharge using PRAM scores (34), assessed hourly in the ED and 4-hourly after admission to an inpatient ward. PRAM scoring was included in the wheeze or asthma care pathway document adapted by the sites for local implementation. In this study, PRAM scoring was considered the standard of care and documented in the pathway document as source data to be used for patient selection (eligibility). If the pathway document could not be implemented at the ED, then the study screening and eligibility process was adapted for the recording and documentation of PRAM scores. After randomization, PRAM scoring was considered study-specific and documented in a clinical data pro forma as source data to be used for treatment monitoring.

In order to ensure consistency across all study sites, intensive training in recording PRAM scores was undertaken. Data quality was reviewed regularly, and training was updated throughout the study. The principal investigator (PI) and local investigators were responsible for promoting the clinical use of the agreed care pathway in advance of the study, for training ED staff on PRAM scoring in the context of the pathway, and for training staff in other departments (eg, HDU and ward) on the use of PRAM. Various training resources were made available to the sites, including locally produced training videos and a web-based PRAM teaching module from Centre Hospitalier Universitaire Sainte-Justine, University of Montréal [53].

Maintaining an adequate level of training for staff regarding the use of PRAM was challenging. Only one of the sites had staff who were familiar with PRAM scoring, and one of the items (palpable scalene muscle contraction) required specific training. Clinical staff had a range of opinions regarding the clinical value of asthma severity scores, and high staff turnover meant that training had to be revisited at frequent intervals.

### Safety Reporting

#### Overview

Section 7 of the study protocol in Multimedia Appendix 1 provides standard definitions of safety reporting technology appropriate for trials other than Clinical Trials of Investigational Medicinal Products, including adverse event (AE), adverse reaction, serious AE (SAE), serious adverse reaction (SAR), and suspected unexpected SAR, and details of the safety reporting procedures (actions and required timelines) used in this study.

#### Recording of AEs

The operational definitions of the AEs collected from randomization to hospital discharge are given in Textbox 10 and include two types of events:

1. Air leaks: ASA is a well-recognized risk factor for air leak. Higher HiFlo rates (>2 L/kg) can mimic the effects of CPAP, which is a theoretical additional risk factor for air leak. This study used more conservative flow levels, previously used safely in HiFlo therapy in patients with asthma [23]; therefore, the risk of air leaks was regarded as low. Air leaks in any of the following 3 manifestations were regarded as an SAE and prompted immediate reporting: pneumothorax, pneumomediastinum, or subcutaneous emphysema.
2. Standard treatment–related AE: Vomiting, tachycardia, tremor, lactic acidosis, and others are known potential side effects of intravenous or inhaled bronchodilators that could be seen in both the HiFlo and standard care groups. Documentation of their occurrences was needed to evaluate the candidate secondary outcome measures (treatment-related side effects). If serious, they could be reported as SAEs.

**Definitions of adverse events.**

1. Pneumothorax: single episode to be reported per patient.
2. Pneumomediastinum: single episode to be reported per patient.
3. Subcutaneous emphysema: single episode to be reported per patient.
4. Vomiting: a period of sequential vomiting is considered a single episode, but if there is a pause of ≥4 hours, then the next occurrence is the start of a new episode.
5. Tachycardia (heart rate ≥160 in children aged 2 to 4 years and ≥140 in those aged 5 to 11 years): an episode lasts for as long as the patient is in tachycardic range, but if there is a pause of ≥4 hours, then the next occurrence is the start of a new episode.
6. Tremor: an episode lasts as long as any tremor is present, but if there is a pause of ≥4 hours, then the next occurrence is the start of a new episode.
7. Lactic acidosis (venous or capillary lactate >2.2 mmol/L or arterial lactate >1.6 mmol/L): an episode starts every time an abnormal laboratory value is detected. The episode lactate value is recorded.
8. Hypokalemia (potassium <3.5 mmol/L): an episode starts every time an abnormal laboratory value is detected.
9. Sedation received to tolerate high-flow humidified oxygen (HiFlo) therapy: single episode to be reported per patient.
10. Patient unable to tolerate HiFlo therapy (HiFlo discontinued): single episode to be reported per patient.
11. Other: any other untoward medical occurrence or serious adverse event in a study participant.

#### Responsibilities of Safety Reporting

The PI at each site was responsible for reporting any SAE or SAR to the clinical trials unit (CTU). The trial manager (TM) was responsible for ensuring that all SAE and SAR reports were complete and accurate and for following up with the research teams to ensure this. The TM was responsible for maintaining and updating all the SAE and SAR records required for reporting to the sponsor and the Research Ethics Committee (REC). All AEs and SAEs were monitored by the TM at the CTU and reported and reviewed at the trial steering committee (TSC) meetings.

### Statistics and Data Analysis

#### Statistical Analysis Plan

Participant flow through the trial will be represented in a CONSORT flowchart (Figure 1) according to the CONSORT extension for pilot and feasibility trials [41]. The available cases will be analyzed, following the intention-to-treat principles. Normally distributed variables will be summarized by means and SDs, skewed continuous variables by medians and IQRs, and categorical variables by frequencies and percentages. The difference in means between the trial arms for the primary and secondary outcomes will be estimated, together with bootstrapped 95% CIs. All analyses will be conducted using Stata (version 18; StataCorp LLC).

#### Qualitative Data Analysis

The interview transcripts will be anonymized and transcribed verbatim. Thematic content analysis will be performed on the interview transcripts based on a 14-stage structured approach [42]. The initial codes will be semantically clustered into subthemes, and finally, these subthemes will be clustered into main themes. The final thematic structure will be described and supported with illustrative interview quotes.

#### Subgroup Analyses and Participant Population

Subgroup analysis will be limited to 3 variables on which randomization was stratified: site, age, and severity of acute episode. The analysis will be conducted on an intention-to-treat basis; all the recruited participants with consent received will be included in the analysis. In addition, we will examine the screening logs at the sites to identify factors involved in the failure to recruit, which may be relevant to the design of the full RCT. Furthermore, per-protocol analysis will be performed, in which deviation from the trial protocol will result in exclusion from data analysis from that point of protocol deviation onward. Examples of protocol deviations include the following:

- A child is randomized to the HiFlo therapy arm, but for logistical reasons (eg, no equipment is available), this therapy never commenced.
- A child in the HiFlo arm is commenced on therapy, which is later discontinued or changed to another modality because of transfer to a ward area that is unable to provide this care.

The per-protocol analysis will be interpreted cautiously because of the small sample size.

### Data Management

A research team pack was used to ensure accurate data collection and included clinical observation sheets and case report forms to record the outcome data. Pseudonymized data were electronically entered by trained research nurses at each site onto a web-based, password-protected data management system (REDCap; Research Electronic Data Capture; Vanderbilt University) designed by a data manager from the Brighton and Sussex CTU (BSCTU). The data manager oversaw data quality and ensured that the database was ready for analysis. Once the data had been cleaned and the database locked, they were transferred securely to the trial statistician for descriptive analysis by the trial arm.

It was agreed that all investigators and trial site staff must comply with the requirements of the General Data Protection Regulation 2018 guidance for researchers—Health Research Authority (HRA) [54]. A specific data management plan and a monitoring plan were developed for this study (available from BSCTU). Archiving will be authorized by the sponsor following the submission of the end-of-trial report. All essential documents will be archived for a minimum of 5 years after trial completion. Destruction of essential documents will require authorization from the sponsor.

### Trial Management and Monitoring

The BSCTU oversaw the management of the study. A TM worked closely with the chief investigator (CI) and research team to ensure that the timelines were met, recruitment was tracked, and remote monitoring was undertaken for quality assurance. The TM supported the setup of the sites, ensuring that all documentation and processes were in line with the research governance and HRA processes. Monthly trial management group (TMG) meetings with the CI, DM, statistician, PIs, and research nurses from the sites were conducted to oversee the study’s progress.

The TSC consisted of the TMG and 3 independent members (a lay member—parent of a child with asthma, a pediatrician with relevant expertise, and a statistician). The TSC reviewed the reports from the TMG and met to oversee the overall progress. With its independent membership, the TSC also reviewed the data and safety issues, fulfilling the role of this feasibility trial of a data and safety monitoring board. Financial management for the study was overseen by the TM with institutional supervision by the head of research at the UHSx NHS Foundation Trust.

### Ethical Considerations

The study protocol and all applicable documents and amendments were approved by the West Midlands–Solihull Research Ethics Committee (REC) and the Health Research Authority (HRA) according to applicable regulations (REC reference: 19/WM/0219, IRAS: 261627). This study was registered with International Standard Randomised Controlled Trial Number registry (ISRCTN78297040) [55]. The HiFlo ASA project proposal was successful in competition 35 of the NIHR Research for Patient Benefit Program after 2 stages of independent peer review by the program’s designated expert advisory panel. The study protocol for this feasibility study was further developed and discussed by researchers from the participating centers, with the involvement of the BSCTU.

### Dissemination

In this feasibility trial, the important aspects of dissemination concern the use of trial data in designing a full RCT and preparing an application to fund it. Therefore, dissemination will be principally among the study team, the PPI groups involved, and the stakeholders (both professional and patient or parent groups) to be involved in the full multicenter RCT. We intend to publish a protocol for this feasibility trial.

### Trial Sponsorship

The sponsor was responsible for ensuring that the trial was being conducted under appropriate governance. The sponsor is the UHSx NHS Foundation Trust.

### Committees

The composition, roles, and responsibilities of the TMG, TSC, and LEAP are described in the *General Information* section (part 6) at the beginning of the study protocol in Multimedia Appendix 1.

### PPI Group

PPI was sought at different stages in the development of the study, with a plan for continuing input during the research and its dissemination.

The detailed involvement of the following PPI groups is described in section 11.3 of the study protocol in Multimedia Appendix 1:

- Two local patient groups from Kent, Surrey, and Sussex (KSS), funded by the NIHR, participated in the trial design and will be informed of the findings of the study: KSS young people’s advisory group and KSS parent and carer advisory group (KSS, Generation R).
- A separate NIHR research support grant enabled the creation of a LEAP—a group of 6 to 8 parents whose young children have been admitted with ASA, together with 4 children with experience of ASA—to provide disease-specific PPI into the study.

## Results

The trial was opened to recruitment at the lead site in February 2020, but a month later, the COVID-19 pandemic reached the United Kingdom, and the study was put on hold in March 2020. There was a 15-month pause because of a combination of factors, including general concerns about face-to-face research, specific concerns about HiFlo therapy as an *aerosol-generating procedure*, and redeployment of research staff to clinical duties during the pandemic. The trial was reopened at the lead site in July 2021 and opened at the other 3 sites between August and December 2022. The follow-up was completed in July 2023. The results are currently being analyzed and will be reported separately.

## Discussion

This paper describes the protocol for a multicenter feasibility RCT of 50 children that has been successfully executed at 4 sites in the United Kingdom. The trial aimed to establish whether a full RCT of early HiFlo therapy in ASA can be conducted successfully and safely and whether recruitment for such a trial, using deferred consent, is practicable in children aged 2 to 11 years presenting to hospital with ASA. If it is determined that a definitive RCT is feasible, the data from the study will inform the outcome measures and sample size needed for adequate power.

A total of 2 previous pilot RCTs of HiFlo therapy in acute asthma have been published [27,28], but neither provided the feasibility data required to design a definitive RCT to assess the clinical effectiveness. Both studies used an asthma severity score as the main outcome measure, but neither the *Pulmonary Score* used by Ballestero et al [27] nor the *Pulmonary Index Score* used by Gauto Benítez et al [28] were referenced, and it is unclear what any differences in these outcomes would mean for clinical management. In this protocol, we chose 2 candidate primary outcome measures that had been determined by clinicians and by parents and children to be meaningful and likely to impact practice. Both the need for escalation of therapy owing to treatment failure and the time to reach readiness for discharge are important in determining resource use and care costs.

We used an asthma scoring system and selected PRAM because it was the only asthma scoring system validated across the entire age range of 2 to 11 years. At least 17 different asthma severity scores have been published, but few EDs in the United Kingdom regularly use any of them as part of routine clinical practice [56], and only one of our sites had any prior familiarity with PRAM. The study protocol used the PRAM score in 2 ways: as one of the entry criteria (PRAM score ≥5 after first-line treatment) and as a candidate (secondary) outcome measure (time to achieve a PRAM score of ≤3). As noted above, implementing PRAM at the study sites and maintaining PRAM competency, despite high staff turnover, required considerable input of training resources. The advantage of using a validated asthma score as an entry criterion was that it standardized the severity of acute asthma in children entering the study, allowed comparison with other acute asthma trials [36], and allowed us to stratify randomization by asthma severity at entry. A potential disadvantage was that it risked reducing out-of-hours recruitment owing to PRAM-trained staff not being available.

As with many clinical trials since 2019, the conduct of this study was severely affected by the COVID-19 pandemic. It was necessary to pause recruitment for a prolonged period because of general logistic issues affecting all clinical research and specific concerns relating to the theoretical risks of aerosol generation and COVID-19 transmission from HiFlo therapy. The experience gained by the research team (both in adapting trial procedures and disseminating new research findings on aerosol generation) to allow the successful completion of the trial will be valuable in planning a definitive RCT.

## Appendix

Multimedia Appendix 1 Full study protocol.

Multimedia Appendix 2 Peer-review report from the National Institute for Health Research.

## Abbreviations

AE: adverse event
ASA: acute severe asthma
BSCTU: Brighton and Sussex clinical trials unit
CONSORT: Consolidated Standards of Reporting Trials
CPAP: continuous positive airway pressure
CTU: clinical trials unit
ED: emergency department
FiO_2_: fraction of inspired oxygen
HDU: high-dependency unit
HiFlo: high-flow humidified oxygen
HRA: Health Research Authority
KSS: Kent, Surrey, and Sussex
LEAP: Lived Experience Advisory Panel
NIHR: National Institute for Health and Care Research
PI: principal investigator
PPI: patient and public Involvement
PRAM: Preschool Respiratory Assessment Measure
RCT: randomized controlled trial
REC: Research Ethics Committee
REDCap: Research Electronic Data Capture
SAE: serious adverse event
SAR: serious adverse reaction
TM: trial manager
TMG: trial management group
TSC: trial steering committee
UHSx: University Hospitals Sussex
