# Supplementary material for: High-Flow Humidified Oxygen as an Early Intervention in Children With Acute Severe Asthma: Protocol for a Feasibility Randomized Controlled Trial
Source: JMIR Res Protoc. 2024 Mar 28;13:e54081. doi: 10.2196/54081 (PMC11009849; doi:10.2196/54081)
Supplement: Multimedia Appendix 1 [file resprot_v13i1e54081_app1.pdf]

High Flow humidified oxygen as an early intervention in children with Acute Severe Asthma: a feasibility study

FULL/LONG TITLE OF THE TRIAL

High Flow humidified oxygen as an early intervention in children with Acute Severe Asthma: a feasibility study.

SHORT TRIAL TITLE / ACRONYM

HiFlo ASA

PROTOCOL VERSION NUMBER AND DATE

Version 4, 10 August 2023

RESEARCH REFERENCE NUMBERS

**IRAS Number:**	**261627**
**FUNDERS Number:**	PB-PG-1217-20024

SIGNATURE PAGE

The undersigned confirm that the following protocol has been agreed and accepted and that the Chief Investigator agrees to conduct the trial in compliance with the approved protocol and will adhere to the principles outlined in the Declaration of Helsinki, GCP guidelines, the Sponsor's (and any other relevant) SOPs, and other regulatory requirements.

I agree to ensure that the confidential information contained in this document will not be used for any other purpose other than the evaluation or conduct of the clinical investigation without the prior written consent of the Sponsor

I also confirm that I will make the findings of the trial publically available through publication or other dissemination tools without any unnecessary delay and that an honest accurate and transparent account of the trial will be given; and that any discrepancies and serious breaches of GCP from the trial as planned in this protocol will be explained.

Chief Investigator:
Signature:
....
Name: (please print):
....

Date: ....../....../......

Statistician:
Signature:
....
Name: (please print):
....

Date: ....../....../......

KEY TRIAL CONTACTS

Chief Investigator	Professor Paul Seddon<br>paul.seddon@nhs.net
Trial Manager	Dr Hector Rojas<br>h.rojas@nhs.net
Sponsor	University Hospitals Sussex NHS Foundation Trust<br>Representative: Mr Scott Harfield<br>01273 696 955 extension: 7497
Funder(s)	NIHR RfPB<br>Grant: PB-PG-1217-20024<br>Contact: laura.tornatore@nihr.ac.uk
Clinical Trials Unit	Brighton & Sussex Clinical Trials Unit<br>Room 204 Bevendean House<br>University of Brighton<br>Falmer, BN1 9PH<br>**bsctu@bsms.ac.uk**
Key Protocol Contributors	Paul Seddon<br>Graham Roberts<br>Akshat Kapur<br>Atul Gupta<br>John Pappachan<br>Michaela Lazner<br>Fleur Cantle<br>Jane Bayreuther<br>Christina Jones<br>Stephen Bremner<br>Hector Rojas<br>Amy Arbon
Statistician	Dr Stephen Bremner
Committees	Trial Management Group (TMG)<br>Trial Steering Committee (TSC)<br>Lived Experience Advisory Panel (LEAP)

i. LIST of CONTENTS

**GENERAL INFORMATION**	**Page No.**
TITLE PAGE AND RESEARCH REFERENCE NUMBERS	1
SIGNATURE PAGE	2
KEY TRIAL CONTACTS	3
i. LIST of CONTENTS	4
ii. LIST OF ABBREVIATIONS	5
iii. TRIAL SUMMARY	6
iv. FUNDING	6
v. ROLE OF SPONSOR AND FUNDER	7
vi. ROLES & RESPONSIBILITIES OF TRIAL MANAGEMENT COMMITTEES, GROUPS AND INDIVIDUALS	7
vii. PROTOCOL CONTRIBUTORS	8
viii. KEYWORDS	8
ix. CONSORT DIAGRAM	9
**SECTION**	
1. BACKGROUND AND RATIONALE	10
2. OBJECTIVES AND OUTCOME MEASURES/ENDPOINTS	12
3. TRIAL DESIGN	14
4. TRIAL SETTING	14
5. PARTICIPANT ELIGIBILITY CRITERIA	15
6. TRIAL PROCEDURES	16
7. SAFETY REPORTING	24
8. STATISTICS AND DATA ANALYSIS	26
9. DATA MANAGEMENT	27
10. TRIAL MANAGEMENT, MONITORING, AUDIT & INSPECTION	28
11 ETHICAL AND REGULATORY CONSIDERATIONS	28
12. DISSEMINATION POLICY	31
13. REFERENCES	32
14. APPENDICES	34

ii. LIST OF ABBREVIATIONS

AE	Adverse Event
AR	Adverse Reaction
ASA	Acute Severe Asthma
BSCTU	Brighton & Sussex Clinical Trials Unit
CI	Chief Investigator
CRF	Case Report Form
CTU	Clinical Trials Unit
CPAP	Continuous Positive Airway Pressure
DM	Data Manager
DMC	Data Monitoring Committee
ED	Emergency department (paediatric)
FiO_2	Fraction of Inspired Oxygen
GCP	Good Clinical Practice
ICF	Informed Consent Form
ICH	International Council for Harmonisation of Technical Requirements for Pharmaceuticals for Human Use
ISF	Investigator Site File (This forms part of the TMF)
ISRCTN	International Standard Randomised Controlled Trials Number
NHS R&D	National Health Service Research & Development
NIMP	Non-Investigational Medicinal Product
pCO_2	Partial Pressure of Carbon Dioxide
PI	Principal Investigator
PIS	Participant Information Sheet
PPI	Patient and Public Involvement
PRAM	Paediatric Respiratory Assessment Measure
QA	Quality Assurance
QC	Quality Control
QP	Qualified Person
RCT	Randomised Control Trial
REC	Research Ethics Committee
RR	Respiratory Rate
RSI	Reference Safety Information
SaO_2	Oxygen Saturation
SAE	Serious Adverse Event
SAR	Serious Adverse Reaction
SDV	Source Data Verification
SOP	Standard Operating Procedure
SmPC	Summary of Product Characteristics
SSI	Site Specific Information
SUSAR	Suspected Unexpected Serious Adverse Reaction
TMF	Trial Master File
TMG	Trial Management Group
TSC	Trial Steering Committee
UHSussex	University Hospitals Sussex NHS Foundation Trust
YPAG	Young Persons (Research) Advisory Group (Kent Surrey & Sussex)

iii. TRIAL SUMMARY

Trial Title	High Flow humidified oxygen as an early intervention in children with Acute Severe Asthma: a feasibility study	
Short Title	HiFlo ASA	
Trial Design	Feasibility randomised controlled	
Trial Participants	Children age 2 to 11 years	
Planned Sample Size	70	
Intervention duration	Until participant is discharged from hospital	
Follow up duration	N/A	
Planned Trial Period	During inpatient stay	
	Objectives	Outcome Measures
Primary Feasibility Objectives and Outcome Measures	1. To evaluate enrolment rates	- Proportion of enrolled children (i.e., randomised) amongst eligible patients with ASA
	2. To evaluate deferred consent rates	- Proportion of children with signed deferred consent amongst those enrolled into the study
	3. To assess feasibility of recording candidate primary outcome measures	- Proportion of data collection complete per participant
	4. To estimate the variability of candidate primary outcome measures	- Summary statistics for candidate primary outcomes
	5. To determine design characteristics for a subsequent definitive study	- Proposed design, sample size and number of centres for a definitive study
	6. To assess the acceptability of HiFlo and the deferred consent model to children, parents and staff	- Satisfaction ratings on exit questionnaire

iv. FUNDING AND SUPPORT IN KIND

**FUNDER(S)**<br>(Names and contact details of ALL organisations providing funding and/or support in kind for this trial)	**FINANCIAL AND NON FINANCIALSUPPORT GIVEN**
NIHR RfPB	**Grant:** PB-PG-1217-20024<br>£249,941.00
Vapotherm	Manufacturer of portable and fixed HiFlo equipment (Vapotherm) units and consumables to make this intervention available cost-free in all 3 EDs for the duration of the study.

v. ROLE OF TRIAL SPONSOR AND FUNDER

Trial funder (NIHR RfPB) : to provide funding to enable the trial to occur, to ensure that timelines are being met and that appropriate dissemination occurs.

Trial sponsor: to ensure that the trial is being conducted with approriate governance.

vi. ROLES AND RESPONSIBILITIES OF TRIAL MANAGEMENT COMMITEES/GROUPS & INDIVIDUALS

- **Trial Management Group (TMG)**

  The TMG will consist of the Chief Investigator (CI), CTU operational manager, trial manager (TM), data manager (DM), senior statistician, Principal Ivestigators and research nurses from the sites.

  The TMG will be responsible for the trial set-up, the day-to-day running of the trial and the release of any trial results or publications according to the BSCTU SOPs. The TM will be involved in setting up monthly TMG meetings which will oversee the management and conduct of the study. Recruitment and data updates will be discussed to highlight any issues and to ensure they can be resolved in a timely manner.

- **Trial Steering Committee (TSC)**

  The TSC will consist of the TMG and the trial co-investigators, together with 3 independent members (a lay member - parent of a child with asthma, a paediatrician with relevant expertise, and a statistician).

  The TSC will meet every 6 months, will receive reports from the TMG and will oversee the progress of the study. With its independent membership it will also review the data and safety issues, fulfilling the role for this feasibility trial of a data and safety monitoring board (DSMB). Financial management for the study will be overseen by the TM with oversight for the project by Head of the Research Department at UHSussex (Scott Harfield).

  The TSC will have oversight of the trial conduct. The Committee's terms of reference, roles and responsibilities will be defined in a charter in accordance with the relevant BSCTU SOP.

- **Lived Experience Advisory Panel (LEAP)**

  The LEAP will consist of 8 parents whose young children have been admitted with ASA, together with four children with experience of Acute Severe Asthma (ASA).

  The LEAP will meet every 6 months and will provide disease-specific input into the study. Their role and involvement is described in section 11.3 below - Patient and Public Involvement (PPI).

vii. PROTOCOL CONTRIBUTORS

All the protocol contributors named in the table at the top of the form have contributed to the development of the protocol in a series of investigator meetings.

PPI input has been provided by the Kent Surrey and Sussex Young Persons (Research) Advisory Group (YPAG) who have had significant influence on the protocol design, especially in the area or recruitment and provision of participant information.

viii. KEY WORDS:

Asthma, management, child, high flow humidified oxygen

ix. CONSORT DIAGRAM(1)

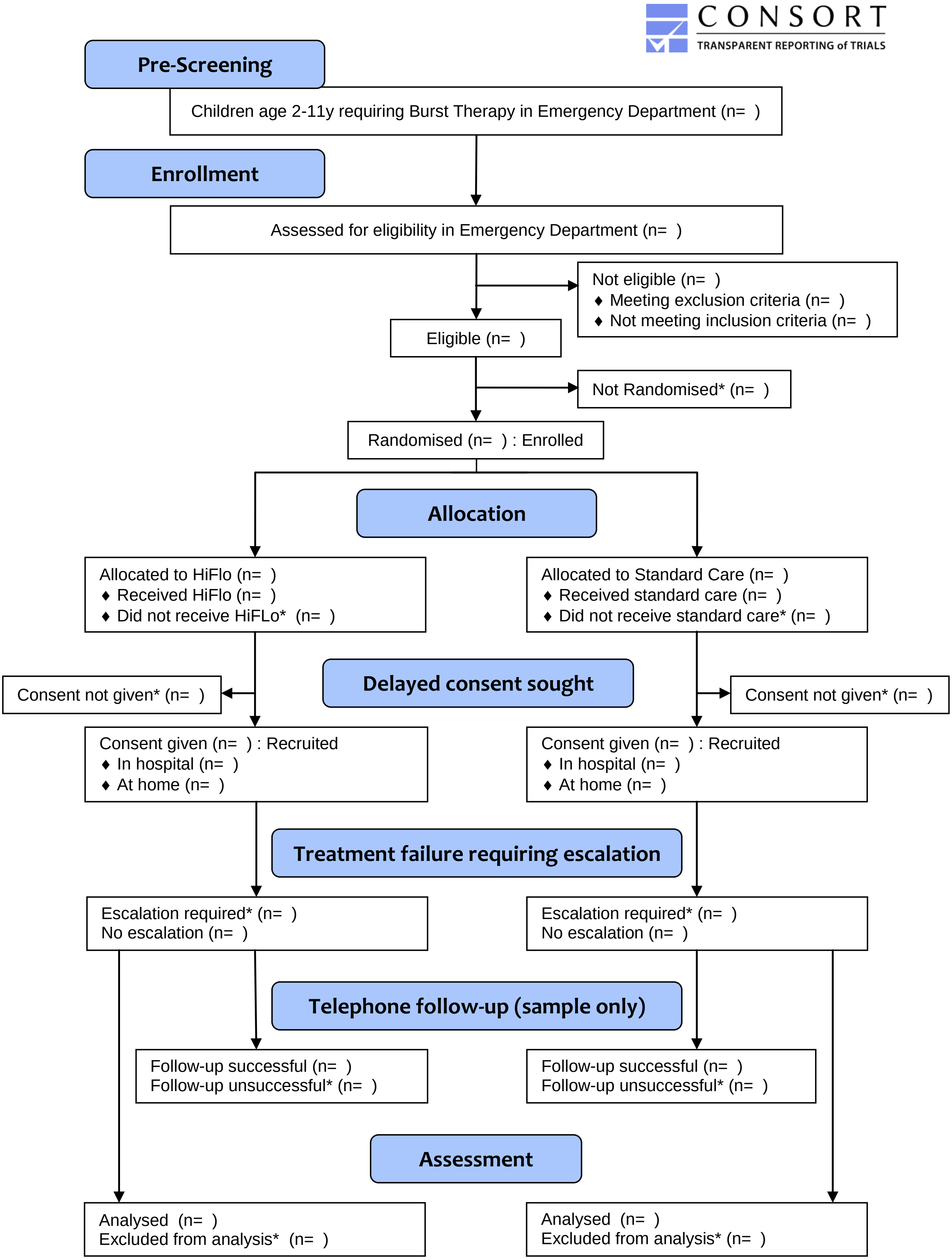

*Record reasons

1. BACKGROUND AND RATIONALE

Asthma is a common chronic disorder of reversible airway obstruction, affecting one in 11 children in the UK(2). The condition is characterised by bronchial smooth muscle contraction, airway inflammation and increased airway secretions(3). Children with asthma are prone to episodes of acute severe airway obstruction characterised by wheeze and increased work of breathing. Many preschool children, not yet diagnosed with asthma, are admitted to hospital with episodes of acute severe wheeze. They present identically, and are treated in the same way, as older children with diagnosed asthma, although they can be less responsive to therapy(4). Throughout this application we will therefore term the problem 'acute severe asthma' (ASA), whether or not children presenting with acute wheeze have an established diagnosis of asthma.

Therapy for ASA is directed at 1) relieving bronchoconstriction with bronchodilators, 2) decreasing airway inflammation with corticosteroids, and 3) clearing airway secretions - not allowing these to become thick and block the airways. Standard first-line emergency treatment(5) for ASA in children starts with 'burst therapy' in the first hour (3 doses of high dose inhaled salbutamol, sometimes with inhaled ipratropium, via a spacer device or nebuliser) plus oral corticosteroids. During the next 1-4 hours many children improve clinically and may be discharged. However, some children fail to respond to standard therapy, and require hospital admission for more intensive, second-line treatment: without effective treatment these children are at risk of fatigue, respiratory failure and death(6). Second-line treatment commonly includes intravenous bronchodilators (one or more of: aminophylline, salbutamol and magnesium sulphate). However, the evidence for the efficacy of such treatments is limited and inconsistent(5), with frequent adverse effects including tachycardia, jitteriness, tremor, palpitations, nausea, vomiting, elevated lactate and hypokalaemia(7). These adverse effects can cause considerable distress to the child and her/his family. Current UK guidelines(5) give little guidance (due to scarcity of evidence) as to which second-line treatment clinicians should use.

There is therefore a need to investigate other options for treating ASA in order to improve the effectiveness of treatment and reduce adverse effects. High flow, highly humidified oxygen therapy (HiFlo) is an innovative health care technology which supports breathing by supplying a warm, humidified air/oxygen mixture at high flow rates(8). This technology has already shown promising results in other acute respiratory conditions in children(8) - see section 3

In the UK in 2011-12, asthma care costs were estimated at over £1.1 billion, with hospital episodes alone accounting for over £90 million(9). ASA is a leading cause of hospital attendance in children, accounting for up to 7% of all paediatric emergency visits(10) and 8.5% of paediatric admissions from emergency departments(11) - the commonest single cause. In the UK, a child is admitted to hospital with acute asthma every 20 minutes(2). Episodes of ASA can disrupt family life, cause school and parental work absence, and hold back academic and social development(12). Progress against key asthma outcomes (including asthma deaths) appears to have stalled over the last 10 years(6). In the UK in 2016 there were 1410 deaths from asthma, compared to 1369 in 2001(2) - with UK populations for those years of 65 million and 59 million respectively, and no significant change in asthma prevalence. A confidential enquiry into UK asthma deaths in 2012, including 28 in childhood, found that almost all could have been prevented(6).

Childhood ASA therefore has important impacts on healthcare costs and quality of life, and presents a risk to life itself. Our proposed research seeks to assess the impact of a novel therapy which has the

potential to treat ASA more effectively and reduce hospital stay and intensive care admissions. This study is timely, as HiFlo is already being adopted widely for bronchiolitis in infants(13) but without convincing published data on its efficacy or health economic benefit in this clinical setting(14). If HiFlo in ASA is not evaluated objectively, there is a risk that a treatment without proven benefit (but with significant costs) may drift into widespread practice.

HiFlo is a health care technology which supports breathing by supplying high flow, warm, humidified air/oxygen mixture via fine, soft nasal cannulae(8). Traditionally, oxygen therapy in asthma and other respiratory diseases has used cold, unhumidified oxygen direct from a cylinder or wall outlet. Although this is helpful in improving oxygenation, it is uncomfortable for patients and causes drying and cooling of the nose and mouth, and potentially of the lower airways - this can cause worsening of airway obstruction and even airway damage. For these reasons, unmodified oxygen therapy can only be delivered at very low flow rates. With HiFlo technology, the air/oxygen percentage mix can be varied, it is warmed to body temperature, and is delivered at 100% humidity. As a result much higher flows can be delivered without discomfort or adverse effects on the airways.

There is now considerable experience in the use of this technology both in adults and children(8). The majority of the clinical experience and clinical evidence for the efficacy of HiFlo in the paediatric population is derived from studies performed in preterm neonates with surfactant deficiency. In this population HiFlo appears to be as effective as continuous positive airway pressure (CPAP) and has become a standard therapy(15). The physiological basis of its effectiveness is unclear(8): HiFlo itself may generate CPAP(16), but it may also reduce nasopharyngeal deadspace, reduce upper airway resistance, and reduce the metabolic demand required to humidify inspired gases(17).

Over the past decade there has been increasing use of HiFlo in infants with acute bronchiolitis(18). A recent audit in one of our units showed that use of HiFlo in bronchiolitis appeared to be safe, and was rapidly replacing other forms of non-invasive support (Derrick R et al, presented at ESPNIC, Lisbon 2017). Retrospective studies have suggested that introducing HiFlo for acute bronchiolitis is associated with reduced need for intubation(18, 19). Prospective trials comparing HiFlo with standard bronchiolitis therapy (low flow 100% oxygen) have shown improved oxygen saturation levels(20), fewer treatment failures(21, 22), and a non-significant trend to faster weaning from oxygen(21). A physiological study has shown that, as in preterm neonates, HiFlo does indeed generate measureable CPAP(23). A Cochrane systematic review has concluded that HiFlo is feasible and well tolerated in bronchiolitis, but that further evidence for its effectiveness is needed(14).

There have so far been no substantial RCTs of HiFlo in children with ASA. The pathophysiology of ASA is very different from that seen in bronchiolitis. Bronchiolitis is characterised by more mechanical distal airway obstruction(14), while in ASA bronchial smooth muscle constriction plays a major role(3, 4). A recent retrospective French study of 73 children with ASA in a paediatric intensive care unit(24) demonstrated that HiFlo was feasible and safe, and that blood gases and clinical parameters improved significantly after starting HiFlo. Very recently (March 2018) a Spanish group has published a pilot RCT (62 children) of HiFlo versus conventional oxygen therapy in ASA(25). HiFlo was instituted in children who were already in respiratory failure, and the stated primary outcome measure was a reduction in a clinical asthma score. A higher proportion of children on HiFlo reduced their score by 2 points over the first 2 hours of treatment. These studies were conducted in high dependency healthcare settings, the intervention was applied later than we are proposing, and the focus was on physiological outcomes. Although these two studies are encouraging, they do not provide the

feasibility information required to plan an RCT of the clinical effectiveness of early HiFlo in childhood ASA in the NHS with the focus being on faster recovery to enable a reduction in time to discharge.

2 OBJECTIVES AND OUTCOME MEASURES/ENDPOINTS

The ***underlying hypothesis*** is that **early** HiFlo in ASA will reduce the need for more invasive treatments , allow faster recovery and discharge from hospital, and in both these ways reduce distress to children and their families.

The **aim** of this feasibility study is to establish whether a full RCT of early HiFlo in ASA can be conducted successfully and safely; and whether recruitment to such trial, using deferred consent, is practicable in children aged 2 to 11 years presenting to hospital with ASA.

2.1 Primary Feasibility Objectives and Outcome Measures

Six objectives and associated outcome measures, shown in Table 1 below, were established to help determine the feasibility of progressing to a full RCT.

Table 1 – Primary feasibility objectives and outcome measures

Feasibility Objectives	Feasibility Outcome Measures	Timepoint of evaluation
1. To evaluate enrolment rates	Proportion of enrolled (i.e., randomised) children amongst eligible patients with ASA	Enrolment
2. To evaluate deferred consent rates	Proportion of children with signed deferred consent amongst those enrolled into the study	Deferred consent
3. To assess feasibility of recording candidate primary outcome measures	Proportion of data collection complete per participant, for candidate primary outcome measures listed in section 2.2	Discharge
4. To estimate the variability of candidate primary outcome measures	Summary statistics for candidate primary outcome measures listed in section 2.2	Discharge
5. To determine design characteristics for a subsequent definitive study	Proposed design, sample size and number of centres for a definitive study	End of study
6. To assess the acceptability of HiFlo and the deferred consent model to children, parents and staff.	Satisfaction ratings on end of study questionnaire	Discharge

In order to progress to a full RCT, we would require the following conditions to be met:

1. At least 50% enrolment rate amongst eligible children (feasibility outcome measure 1)

2. At least 70% deferred consent rate(26) (feasibility outcome 2)

3. At least 80% of data collection complete per participant (feasibility outcome 3)

4. Confirmation that predicted sample size, number of centres,enrolment rates and recruitment (enrolment plus deferred consent) rates would allow an appropriately powered RCT to be conducted in the UK over 3 years (feasibility outcome 5).

Discussions with colleagues indicate that at least 15 large UK paediatric centres would be interested in participating in a definitive RCT on this question. The study has been discussed with two relevant research networks: a) the NIHR Children Respiratory and Cystic Fibrosis Clinical Studies Group; b) Paediatric Emergency Research in the UK and Ireland (PERUKI), who have indicated that they will facilitate the process of identifying appropriate centres for the definitive study. PERUKI, a network of research-active paediatric emergency care clinicians, have indicated their support for this feasibility study, and will help the research team in rapidly identifying one or more additional centres for this study should there be unforeseen problems with recruitment (letter attached to application).

2.2 Candidate Primary Outcome Measures

Two candidate primary outcome measures to be recorded and evaluated as part of feasibility objectives 3 and 4:

- Treatment failure needing escalation of therapy as defined in section 6.3.2 below.
- Time (hours) between presentation to ED and meeting hospital discharge criteria as defined in section 6.5.1 below.

Hospital discharge criteria are defined as:

  - The ability of the child to maintain arterial oxygen saturations measured by pulse oximeter (SaO_2) ≥92% without supplemental oxygen or respiratory support, over a 4-hour period

  AND

  - The ability of the child to go for 4 hours minimum between inhaled bronchodilator doses

2.3 Candidate Secondary Outcome Measures

- Time (hours) between presentation to ED and actual hospital discharge (may differ from above for non-clinical reasons)
- Time (hours) between presentation to ED and achieving a Paediatric Respiratory Assessment Measure (PRAM) score ≤ 3
- Time (hours) between presentation to ED and ability to maintain SaO_2 ≥ 92% without supplemental oxygen or respiratory support
- Need for intravenous (IV) bronchodilator therapy
- Duration of IV bronchodilator therapy
- Requirement for non-invasive ventilation
- Requirement for invasive ventilation (intubation)
- Treatment-related adverse effects
  - Intravenous/inhaled bronchodilator related side effects (vomiting, tachycardia, lactic acidosis)
  - Poor compliance with HiFlo

a) number needing to discontinue HiFlo because unable to tolerate
b) number requiring sedation in order to tolerate HiFlo

- Hospital readmission within 48 hours of discharge
- Acceptability and comfort score for treatment during the episode (recorded by end of study questionnaire and by qualitative interview following the episode). These measures have been co-developed with the Lived Experience Advisory Panel in advance of the trial commencing.

3 TRIAL DESIGN

A feasibility randomised controlled trial (RCT) of 70 children (2-11 years) in 3 children's hospitals in the UK. Eligible children will be randomised to intervention (HiFlo) or control (Standard Care) arms (35 in each arm). The trial is designed to generate the data required to plan a definitive RCT that would satisfy the clinical and health economic end points, and the requirements of children, parents, clinicians and NHS England.

The size of the study has been determined by the number of children required to provide an accurate estimate of the variability in the candidate primary outcome measures: recommendations for this vary between 50(27) and 70(28). We have opted for the larger number to allow for a 30% attrition to deferred consent(26). The subsequent definitive RCT will determine whether HiFlo is an effective intervention in ASA.

This study will be pragmatic, and HiFlo will be an add-on to existing therapy in those randomised to the intervention arm. Children will not be denied access to existing standard second line interventions (e.g. intravenous bronchodilators) as a result of participation in the study. The treating clinical team will be allowed to initiate intravenous bronchodilators as clinically indicated in either treatment arm. In children randomised to the HiFlo arm, HiFlo will be commenced as soon as possible after randomisation, and should be the next treatment initiated rather than IV bronchodilator. If equipment is not available to allow HiFlo to be commenced within 30 minutes of randomisation, the child should not be recruited to the study, and this should be recorded in the screening log. As existing treatment guidelines(5) make no specific recommendations, and because the choice of intravenous bronchodilators is physician-dependent across our three institutions, the study protocol will be physician-led, and will not specify which intravenous bronchodilator is initiated first. Equally, if a child randomised to standard care is failing to respond, as defined by preset criteria, the clinical team can opt to initiate HiFlo as rescue therapy - the child would remain in the study on an intention to treat basis. Reasons for discontinuing the intervention prematurely or other protocol violations will be clearly recorded.

4 TRIAL SETTING

- multicentre
- screening, recruitment and randomisation will take place in the relevant emergency ED
- a deferred consent model will be used to avoid delay in treatment and minimise distress to families who present to the ED with acutely unwell children. Informed consent will not be sought prior to randomisation, but parents will be approached for informed consent within a maximum of 72 hours of randomisation, once their child's condition is more stable. If consent is declined, the child will then exit the study.

5 PARTICIPANT ELIGIBILITY CRITERIA

Children aged 2-11 years will be eligible if they present to hospital with ASA and fail to respond to standard first line therapy (high-dose inhaled bronchodilators).

5.1 Inclusion criteria

- Participants having an acceptable individual capable of giving consent on the participant's behalf (e.g. parent or guardian of a child under 16 years of age)
- Age 2-11 years
- ASA, defined as respiratory distress combined with wheeze on auscultation (a formal preceding diagnosis of asthma is not necessary)
- Failure to respond to standard initial emergency management(5) with 'burst' therapy (back-to-back 3 consecutive inhaled or nebulised doses of salbutamol with or without the addition of ipratropium bromide over a 1-hour period) plus systemic corticosteroids, with or without subsequent intravenous bronchodilator therapy as deemed appropriate by the treating physician.

Failure to respond will be defined as:

PRAM score of 5 or more, between 1 and 4 hours after starting burst therapy.

PRAM score has been shown to be a good predictor of need for admission and escalation of therapy(35). A child with PRAM score = 5 would typically have moderately increased work of breathing, audible wheeze and oxygen saturation below 92% but above 90%.

5.2 Exclusion criteria

- Clinical/radiological evidence of bacterial pneumonia: fever >38.5 ^{o}C **PLUS** focal signs on auscultation or chest X-ray.
- Signs of impending respiratory failure mandating imminent intubation. These will be at the discretion of the treating clinical team, but would include elevated pCO_2, refractory hypoxaemia and exhaustion.
- Contraindications to use of HiFlo:
  - air leak (pneumothorax, pneumomediastinum or subcutaneous emphysema)
  - decreased level of consciousness - AVPU score P or worse
  - recent (within 6 weeks) bowel surgery
  - intractable vomiting
- Other major respiratory, cardiovascular or neurological condition
- Previous participation in the HiFlo ASA study, during a prior hospital episode

6 TRIAL PROCEDURES

This section describes all procedures and evaluations to be done as part of the trial to support the feasibility objectives, in relation to the established study visits. The timing of procedures at each study visit is described in Appendix 1 - Schedule of Events.

6.1 Screening phase

The participant eligibility screening process will be conducted by ED clinical staff or research nurses. However, only children whose eligibility has been confirmed by the treating clinician can be recruited and randomised into the study.

Posters will be clearly displayed in the ED informing families that the study is taking place, and leaflets giving additional information about the study will be available on request and in display racks.

6.1.1 Participant identification

Children arriving at participating EDs are routinely triaged by an experienced nurse. All children potentially eligible for the study will be identified at this stage and actively screened for inclusion by ED clinical staff or research nurses.

6.1.2 Screening

All children are routinely reviewed clinically during and after completing burst therapy. Experienced ED nurses will identify children who fail to respond to standard first line therapy (high-dose inhaled bronchodilators) as defined in section 5.1 above (i.e. PRAM score 5 or more, 1-4 hours after commencing burst therapy).

If participants are screened but not enroled (not eligible and/ or not randomised) then their anonymised data will be recorded and collated for Consolidated Standards of Reporting Trials (CONSORT) for reporting the generalisability of the results. Screening information will include Trust ID number, age and reasons not eligible for trial participation, or if they are eligible but declined. In this case the chosen anonymisation technique will guarantee that the true identity of individual children cannot be derived from the collected data.

6.2 Enrolment (T0)

If a child meets all inclusion criteria, and has no exclusion criteria, he/she will be recruited into the study and randomised to the intervention (early HiFlo) or control (conventional therapy) arms, ONLY after confirmation of eligibility by the treating clinician.

6.2.1 The randomisation scheme

Randomisation occurs at enrolment and before consent (see deferred consent in section 6.4 below). Randomisation will be stratified by site, age (less than 5, 5 and over) and severity of acute asthma (PRAM score - see section 6.6.3 below - at study entry: less than 8, 8 and above), with an equal ratio between both arms.

Randomisation will be implemented using 'Sealed Envelope' online randomisation software (https://www.sealedenvelope.com/) and will be conducted by a delegated member of the research team. A copy of the treatment allocation will be sent by email to the person conducting the randomisation, and to the trial manager.

A screening number will be manually allocated prior to randomisation and will be used during the randomisation procedure. A study number will be allocated subsequently, but only for patients with signed deferred consent (see section 6.4).

6.3 Treatment phase (T1, T2, T3, T4, T8, T12, T16, T20, T24, ... , TC)

The intervention is an add-on to standard care. The key difference between the groups in the two arms is the early use of HiFlo - ie starting HiFlo as the next measure after failure of burst therapy - and it is this strategy which is being examined.

6.3.1 HiFlo use guidelines

HiFlo is already in use in high-dependency areas in all participating hospitals, so there is widespread familiarity with its use, and further staff training will be undertaken in the setup period before the start of the study.

Use of HiFlo in the study will follow established practice, and will be standardised as far as possible while allowing for clinical judgement. Reasons for escalating and reducing treatment will be recorded in the study pack described below in section 9.1, and these data will help in defining treatment escalation and weaning pathways for the definitive RCT.

HiFlo rate will be commenced at a flow of 2L/kg/minute for the first 10Kg body weight, plus an additional 0.5 flow L/kg/minute for every Kg body weight above 10, to maximum absolute flow of 40 L/minute, and FiO_2 (Fraction of inspired oxygen) adjusted appropriately to maintain SaO_2 (Oxygen Saturation) greater than 92%. At the discretion of the treating clinician, flow can be stepped up to 3 L/Kg/minute but again with a maximum absolute flow of 40 L/min.

6.3.2 Treatment Escalation

The following criteria will be used and recorded for treatment failure needing escalation of therapy:

1) PRAM score rising or not coming down
2) Respiratory rate rising or not coming down
3) Heart rate rising or not coming down
4) Rising FiO_2 or pCO_2
5) Other clinical concern (specified)

Table 2 – Escalations in therapy

HiFlo group	Standard Care group
Commencing intravenous bronchodilator therapy	Commencing HiFlo
Commencing 2nd or 3rd intravenous agents	
Re-escalating inhaled bronchodilator therapy to hourly or more frequent	
Commencing non-invasive ventilation with bi-level positive airway pressure ventilation (BiPAP)	
Intubation for invasive ventilation	

For the candidate primary outcome: "Treatment failure needing escalation of therapy", escalations in therapy are defined in Table 2 above. Pragmatically senior clinicians on duty managing these patients will have discretion to escalate treatment if clinically deemed appropriate and justified, but will be asked to clearly state the reason for escalation. Note that order in which escalations are listed in Table 2 does not imply that they will be implemented in this order - clinicians will be free, for example, to implement HiFlo in the Standard Care group before a 2nd or 3rd IV agent.

For the primary analysis, commencing a first intravenous agent is not categorised as "treatment failure requiring escalation" in the Standard Care group, since starting an intravenous agent would be the standard next step in a child who fails burst therapy.

However, it is possible that some children randomised to standard care may not receive an IV agent straight away. It is also useful to examine whether commencing early HiFlo has an effect on the total burden of invasive treatments required. We therefore intend to carry out a secondary analysis in which escalations of therapy are defined as in Table 3 below:

Table 3 – Escalations in therapy - secondary analysis

HiFlo group	Standard Care group
Commencing 1st, 2nd or 3rd intravenous agents	
	Commencing HiFlo
Re-escalating inhaled bronchodilator therapy to hourly or more frequent	
Commencing non-invasive ventilation with bi-level positive airway pressure ventilation (BiPAP)	
Intubation for invasive ventilation	

6.3.3 Weaning from HiFlo

Weaning from Hiflo will commence once the child is clinically stable, according to standard criteria across the 3 units. Essentially, reduction in FiO_2 will occur first then flow rate will be reduced in stepwise fashion once FiO_2 is consistently 40% or lower. Weaning strategy will adopt the schema previously published in the protocol for the FIRST-ABC study:
https://bmjopen.bmj.com/content/bmjopen/7/6/e016181.full.pdf

6.4 Consent

A deferred consent model will be employed to avoid delay in treatment and minimise distress to families who present to the ED with acutely unwell children. Informed consent will not be sought prior to randomisation, but parents will be approached to seek informed consent once their child's condition is more stable during the treatment phase. This will normally be within 24 hours of randomisation, but in some cases may be up to 72 hours after randomisation. If consent is declined, the child will then exit the study.

6.4.1 The deferred consent process

Parents will only be approached for informed consent by trained staff from the direct care team, who will document the process on the consent tracking form of the Case Report Form (CRF):

- the study will be explained to parents who will be provided with a parent information leaflet

- age appropriate information sheets will be provided to the children
- parents will be given time to read and understand the information
- parents will be given the opportunity to clarify any questions regarding the study and their child participation
- written informed consent will be taken
- copies of consent: one with parents, one in medical notes, one to research team.

In the event that a child is discharged from hospital before parents can be approached for deferred consent, they will be contacted by telephone within 72 hours of randomisation by a trained research nurse, who will explain the study over the phone. Written participant information and consent form will then be sent out by email or post. This model has been used successfully in the EcLIPSE study(29), and feedback from parents has been positive(30).

The information pack sent to parents will include a PIS, a consent form, and an option to actively decline consent for their child's data to be used. Whenever possible, full written consent by email or post will be sought in this way. However, it has been the experience in previous similar studies that parents frequently give verbal consent by telephone but for a variety of reasons do not get round to returning the consent form. If neither written consent nor confirmation of declined consent has been received within 4 weeks of sending out the study information, a further telephone call will be made to seek verbal consent. If neither written nor verbal consent is received, the child's data will not be included in the study analysis.

It is possible, though unlikely, that a child recruited into the study could deteriorate and die before consent has been obtained. In this situation, it is clearly important if possible to include the child's data, but the approach must be sensistive and individualised. A site research team member will obtain information from colleagues and bereavement counsellors to establish the most appropriate research/clinical team member to notify parents of their child's involvement in the study. Deferred consent can be sought from parents following the death of their child and prior to their departure from the hospital; however, it is at the discretion of the site staff to determine if this is appropriate for each individual family. In this situation, a specific Participant Information Sheet and consent form for bereaved parents would be used.

If deferred consent is not sought prior to parents' departure from the hospital, then they will be sent a covering letter, personalised by the most appropriate research/clinical team member, and a copy of the relevant PIS and Consent Form by post four weeks after randomisation. Where possible, the clinical or research team member should already be known to the family. The letter will explain their child's involvement in the study, direct them to the PIS for detailed information on the study and provide telephone contact details if parents wish to discuss the study with a member of the site research team. If parents actively decline, or if there is no response, a further letter will be sent 4

weeks later. If there is again no response, there will be no further contact and their child's data will not be included in the analysis.

All written material presented to the patient and their family will be approved by the Research Ethics Committee (REC) and in compliance with Good Clinical Practice (GCP), local regulatory requirements and legal requirements. There will be plenty of opportunity for potential participants to ask questions.

The Principal Investigator (PI) retains overall responsibility for the conduct of research at their site, this includes the taking of informed consent o f participants at their site. They will ensure that any person delegated responsibility to participate in the informed consent process is duly authorised, trained and competent to participate according to the ethically approved protocol, principles of GCP and Declaration of Helsinki.

6.5 Hospital discharge (TD)

6.5.1 Hospital Discharge Criteria (TC)

We recognise that timing of discharge from hospital is affected by multiple administrative and social factors in addition to the child's medical condition. We have therefore defined criteria of fitness for discharge (at time TC) as a more robust and reproducible candidate primary outcome measure, in addition to actual hospital discharge (at time TD) which is still used as a candidate secondary outcome measure.

Hospital discharge criteria are defined as:

- The ability of the child to maintain arterial oxygen saturations measured by pulse oximeter (SaO_2) ≥92% without supplemental oxygen or respiratory support, over a 4-hour period

AND

- The ability of the child to go for 4 hours minimum between inhaled bronchodilator doses

AND

- These conditions are then maintained continuously until hospital discharge

Operationally, TC - the time of meeting hospital discharge criteria - will be recorded as *the time point 4 hours after the last bronchodilator dose for which the interval from the preceding dose was less than 4 hours, providing no supplemental oxygen has been needed during these 4 hours, and provided there is no re-escalation of therapy (e.g. reinstatement of oxygen therapy or increase in bronchodilator frequency) from then until hospital discharge.*

6.5.2 Quantitative analysis of end of study questionnaire data

The study includes a patient satisfaction questionnaire for all parents and their children to be collected at time of hospital discharge. This is tailored to children or parents with acute severe wheeze, and will measure global satisfaction outcomes.

In addition to the qualitative sub-study proposed below (section 6.7) and in lieu of the fact that we do not know how many parents will consent to be interviewed during the follow-up, we propose the inclusion of a brief patient satisfaction questionnaire for all parents and their children to be collected at time of discharge. Whilst validated measures of satisfaction exist within the emergency department setting, these are not tailored to children or parents with acute severe wheeze.

Therefore, another function of the LEAP and YPAG (see PPI section 11.3) has been to finalise questionnaire items measuring global satisfaction outcomes. Items related to treatment effectiveness, treatment satisfaction, service satisfaction, physical comfort, pain and communication(31) were included to be rated on a Likert or visual analogue scale for parents and using pictographic tools, similar to the FACES pain scale(32) and the Children's Asthma Control Test(33) for children aged 4 years and older.

6.6 Trial assessments

Standard of care is guided by a well defined wheeze / asthma care pathway for children. The pathway includes various observations to aid with treatment decision-making at participating centres. Key observations and assessments relevant to this clinical trial are summarised next.

6.6.1 Physical examination

Includes evaluation of suprasternal retraction, scalene muscle contraction, air entry, wheezing, work of breathing (respiratory distress), chest findings and cardiovascular system.

6.6.2 Vital signs

Vital signs at initial assessment (triage) and during subsequent reassessments include respiratory rate (RR), heart rate (HR), oxygen saturation (SaO_2), capillary refill time (CRT) and temperature (Temp).

6.6.3 PRAM scoring

Progress will be monitored regularly from ED admission until discharge from hospital with PRAM scores (34): https://www.chusj.org/en/soins-services/A/Asthme/Professionnels-de-la-sante/Contexte-de-soins-aigus/Paediatric-Respiratory-Assessment-Measure-(PRAM)) . PRAM score will be assessed hourly in the ED and 4-hourly after admission to an inpatient ward. Assessments required for PRAM scoring involve physical examination and pulse oximetry which are all routine procedures in the participating units.

In order to ensure consistency across all study sites intensive training in recording PRAM score across the sites will be undertaken. Data quality will be reviewed regularly and training updated throughout the study.

In order to determine SaO_2 levels accurately for PRAM score, trained staff will turn FiO_2 down to room air for up to 60 seconds until pulse oximeter reading stabilises and record saturation at that point. If SaO_2 drops below 92%, recommence oxygen immediately and record as "less than 92%".

6.6.4 O_2 requirement

Includes monitoring of O_2 flow and fraction of inspired oxygen (FiO_2).

6.6.5 Blood gases

A proportion of patients may have blood gases measurement performed routinely. Specifically pCO_2 results (if available) will be used for treatment escalation decisions. In children, these will normally be done on capillary or venous blood.

6.7 Qualitative assessments

A qualitative sub-study (Participant and health professional experience) has been incorporated in order to explore both the acceptability of HiFlo compared to conventional therapy and the acceptability of the deferred consent process, amongst parents and health professionals.

Semi-structured qualitative telephone interviews with participants and staff will be conducted for this purpose.

6.7.1 Qualitative outcomes

A final topic guide will be devised with assistance from the Lived Experience Advisory Panel (LEAP) - see PPI section 11.3 below for more detail - and will aim to explore the following questions:

1) How acceptable did parents, children and health professionals find the treatment approach used in this study?

2) What aspects of the treatment and the study more generally worked well?

3) What aspects of the treatment and the study more generally need to be improved?

4) If applicable, how did the treatment approach differ from those experienced in the past?

5) What would participants change about the therapy or study more generally?

6) Were there any outcomes which weren't measured which should have been?

7) What did parents, children and health professionals think about the deferred consent process?

8) What would encourage other parents, children and health professionals to participate in this study?

6.7.2 Recruitment and interview procedure(Parents and health professionals)

Parents will be invited to participate in a semi-structured telephone interview with an experienced qualitative researcher to elicit their views and opinions of the therapy and the study more generally one-two weeks post-discharge (at follow-up).

As focus groups with emergency medicine staff will not be feasible given the acute situation, health professionals involved in the delivery of the study and deferred consent process will be invited to participate in semi-structured telephone interviews. These will be conducted whilst the study is ongoing but once a site has recruited at least 10 participants.

All telephone interviews are expected to last a maximum of 30 minutes, and will be recorded for later transcribing.

To ensure feedback is obtained across sites, a stratified sample will be employed whereby 6 parents and 3 health professionals at different grades (half from each arm of the study and from each site), will be recruited. This will yield 24 parents and 12 health professionals for the qualitative sub-study. Whilst a formal sample size is not appropriate for qualitative research, this number of participants is likely to achieve data saturation (no new themes emerging from consecutive interviews) and high information power due to the dense specificity and narrow aims(35).

6.7.3 Qualitative analysis of interview data

All transcripts will be anonymised and transcribed verbatim. All participants' comments regarding acceptability and feedback will be managed in NVivo software. Thematic content analysis will be performed on the interview transcripts based on the 14 stage structured approach described by Burnard(36). This involves familiarisation with the content of the transcripts, before generating initial codes that stay close to the data. These codes will be semantically clustered into sub-themes, and finally, these sub-themes will be clustered into main themes. The final thematic structure will be

described and supported with illustrative quotes from the interviews. Interviews and analysis will be conducted concurrently so emerging themes can be explored in subsequent interviews. The proposed LEAP (see PPI section 11.3) will be trained on basic qualitative analysis skills in order to support interpretation of the data, a format which the co-applicant has used successfully in a different study of caregivers(37). The use of thematic content analysis will enable us to identify patterns of meaning within and across participants. If a definitive trial is justified, these qualitative results will be instrumental in refining the study protocol.

6.8 Discontinuation of study intervention

Discontinuation from HiFlo does not mean discontinuation from the study, and remaining study procedures should be completed as indicated by the study protocol. If a clinically significant finding is identified after enrolment, the investigator or qualified designee will determine if any change in participant management is needed. Any new clinically relevant finding will be reported as an adverse event (AE).

The criteria for enrolment must be followed explicitly. If a patient who does not meet enrolment criteria is inadvertently enrolled, that patient is to be discontinued from the study intervention but observation should be continued according to the study protocol in order to provide the follow-up data needed for the analysis of the entire intention-to-treat population, to which the patient belongs formally.

In addition, patients will be discontinued from study intervention if the investigator decides that the patient should be withdrawn from early HiFlo treatment. If this decision is made because of a serious AE (SAE), the study intervention is to be discontinued, and appropriate measures are to be taken according to the trial Safety Reporting procedure. The Sponsor or its designee is to be notified immediately.

6.9 Participant discontinuation/withdrawal from the study

A participant may be withdrawn from the study at any time at the request of his/her parent(s), or may be withdrawn at any time at the discretion of the investigator or sponsor for safety or administrative reasons. The participant will remain free to withdraw at any time from the trial without giving reasons and without prejudicing his/her further treatment and must be provided with a contact point where he/she may obtain further information about the trial.

Hospital discharge (TD) data (see Appendix 1 Schedule of Events) should be collected at the time of study discontinuation and the reason for participant discontinuation or withdrawal from the study will be recorded on the early discontinuation form of the CRF. Data and samples collected up to the point of withdrawal can only be used after withdrawal if the participant has consented for this, this must be clarified at the time of withdrawal of consent.

There will be no replacement of subjects who withdraw after they have been enrolled in the study and received the study intervention.

6.10 End of trial

A child is considered to have completed the study if he or she has completed all phases of the study including the last visit or the last scheduled procedure shown in the Schedule of Events, Appendix 1. The end of the trial is defined as the end of participation of the last patient recruited to the study (completion of the last visit or procedure shown in the Schedule of Events in the trial globally).

7 SAFETY REPORTING

7.1 Definitions

Term	Definition
**Adverse Event (AE)**	Any untoward medical occurrence in a patient treated on a study protocol, which does not necessarily have a causal relationship with a study treatment. An AE can therefore be any unfavourable and unintended sign (including an abnormal laboratory finding), symptom or disease temporally associated with the use of a study treatment, whether or not related to that study treatment.
**Adverse Reaction (AR)**	An untoward and unintended response in a participant to an intervention which is related to the administration of that intervention. A causal relationship between the trial intervention and an AE is at least a reasonable possibility, i.e. the relationship cannot be ruled out.<br>All cases judged by either the reporting medically qualified professional or the Sponsor as having a reasonable suspected causal relationship to the intervention qualify as adverse reactions.
**Serious Adverse Event (SAE)**	A serious adverse event is any untoward medical occurrence that:<br>• results in death<br>• is life-threatening<br>• requires inpatient hospitalisation or prolongation of existing hospitalisation<br>• results in persistent or significant disability/incapacity<br>• consists of a congenital anomaly or birth defect<br>Other ‘important medical events’ may also be considered serious if they jeopardise the participant or require an intervention to prevent one of the above consequences.<br>NOTE: The term "life-threatening" in the definition of "serious" refers to an event in which the participant was at risk of death at the time of the event; it does not refer to an event which hypothetically might have caused death if it were more severe.
**Serious Adverse Reaction (SAR)**	An adverse event that is both serious and, in the opinion of the reporting Investigator, believed with reasonable probability to be due to one of the trial interventions, based on the information provided.
**Suspected Unexpected Serious Adverse Reaction (SUSAR)**	A serious adverse reaction, the nature and severity of which is not consistent with the information about the intervention in question set out in the reference safety information, defined in the protocol.

7.2 Recording of Adverse Events

Study documentation on safety reporting will standardise the recording of adverse events including but not limited to the following.

7.2.1 Air leaks

Acute severe asthma itself is a well recognised risk of air leak. Higher HiFlo rates (2L/kg or more) can mimic the effects of continuous positive airway pressure, which is a theoretical risk factor for air leak. In this study we are using more conservative flow levels, previously used safely in HiFlo in asthma(29, 30); so the risk of air leaks is minimal.

Air leaks in any of the following three manifestations will be regarded as a SAE and will prompt immediate reporting: pneumothorax, pneumomediastinum or subcutaneous emphysema.

7.2.2 Standard treatment-related adverse effects

Vomiting, tachycardia and lactic acidosis are known potential side effects of intravenous/inhaled bronchodilators that could be seen in both the HiFlo and the standard care goups. Their occurrences will be documented in the CRF as part of the candidate secondary outcome measures (treatment related side effects). If serious they will be reported as SAEs.

7.3 Summary of reporting action required:

Type of Event	Action Required
Adverse Event	Record on an AE/AR reporting log
Serious Adverse Event	Report within 24 hours to ***BSCTUsafety@bsms.ac.uk***
Adverse Reaction	Record on an AE/AR reporting log
Serious Adverse Reaction	Report within 24 hours to ***BSCTUsafety@bsms.ac.uk***

7.4 Detailed Reporting Procedure for SAEs and SARs:

1. An SAE form must be completed by the local Investigator (as named on the delegation of responsibilities log), with the causality and expectedness of the event clearly documented. In the absence of the responsible investigator the form should be completed and signed by a member of the site trial team. The responsible investigator should subsequently check the SAE form, make changes as appropriate, sign and then send to the Brighton & Sussex CTU as soon as possible. The initial report shall be followed by detailed, written reports as appropriate.
2. Send the SAE form by email to BSCTUsafety@bsms.ac.uk within one working day of the investigator's knowledge of the event. The SAE will then have the causality and expectedness assessed by the study's Chief Investigator acting as Clinical Reviewer.
3. Follow-up: Patients must be followed-up until clinical recovery is complete, or until the event has stabilised. Follow-up should continue after completion of protocol treatment if necessary. The patient must be identified by trial number and date of birth only. The patient's name should not be used on any correspondence.
4. The sponsor will notify the research ethics committee of SUSARs as per the conditions of the favourable opinion.

7.5 Recording and reporting of SUSARs

For this study, only reports of Serious Adverse Events (SAEs) that are:

- **related** to the study (I.e they resulted from administration of any of the research procedures) and
- **unexpected** (i.e not listed in the protocol as an expected occurrence)

(SUSARs) should be submitted to the REC using the HRA Non-CTIMP safety report to REC form.

7.6 Responsibilities of Safety Reporting

The principal investigator at each site will be responsible for reporting any SAE/SARs to the CTU.

The trial manager will be responsible for ensuring any SAE/SAR reports are complete and accurate and will follow up with the research teams to ensure this. The trial manager will maintain and update all SAE/SAR records required for reporting to Sponsor and REC.

Monitoring of all AEs and SAEs will be carried out by the CTU, and they will be reported and reviewed at each TSC meeting.

8 STATISTICS AND DATA ANALYSIS

8.1 Sample size justification

This is a feasibility randomised controlled trial (RCT) of 70 children (2-11 years) in 3 children's hospitals. The size of the study has been determined by the number of children required to provide an accurate estimate of the variability in the candidate primary outcome measures: recommendations for this vary between 50(32) and 70(33). We have opted for the larger number to allow for a 30% attrition to deferred consent(31). The subsequent definitive RCT will determine whether HiFlo is an effective intervention in ASA.

8.2 Planned recruitment rate

The study aims to recruit 70 children, within 18 months, from three collaborating centres.

8.3 Statistical analysis plan

Participant flow through the trial will be represented in a CONSORT flow chart (attached), according to the CONSORT extension for pilot and feasibility trials(37). Available cases will be analysed, following intention to treat principles.

Normally distributed variables will be summarised by means and standard deviations, skewed continuous variables by medians and interquartile ranges and categorical variables by frequencies and percentages. The difference in means between trial arms for the primary and secondary outcomes will be estimated, together with bootstrapped 95% confidence intervals. All analyses will be conducted in Stata, version 15 or higher (StataCorp. 2017. Stata Statistical Software: Release 15. College Station, TX: StataCorp LLC).

8.4 Subgroup analyses

Subgroup analysis will be limited to the three variables on which randomisation was stratified, ie site, age, and severity of acute episode.

8.5 Participant population

Analysis will be by intention to treat - all participants recruited and with consent received will be included in the analysis. In addition, we will look at screening logs at the sites to pick out factors involved in failure to recruit, which may be relevant to the design of the full RCT.

Per protocol analysis will also be performed in which deviation from trial protocol will result in exclusion from analysis of data from that point of protocol deviation onwards. Examples of protocol deviation would include:

- a child is randomised to the HiFlo arm, but for logistical reasons (no equipment available) this is never commenced.

- a child in the HiFlo arm is commenced on therapy which is later discontinued or changed to another modality because of transfer to a ward area which is unable to provide this care.

Interpretation of the per protocol analysis will be cautious due to the small sample size.

8.6 Economic evaluation and cost implications

We will not carry out a formal health economics analysis in this feasibility study. We have taken advice from a health economist (Professor Heather Gage, University of Surrey) and will record data

(and document the ease and accuracy of collecting this data) which would be required to plan a full health economic analysis in the subsequent definitive RCT.

We have secured agreement from a manufacturer of portable HiFlo equipment (Vapotherm) to make this intervention available cost-free in all 3 EDs for the duration of the study. This will allow HiFlo to be instituted without delay following randomisation, and to continue during transfer to the inpatient area. A letter confirming their support is attached.

9 DATA MANAGEMENT

9.1 Data collection tools and source document identification

In order to ensure accurate data collection, a study pack will be employed which will function as the clinical case notes for all children entering the study. The pack will include clinical observation sheets to facilitate clear recording of important outcome data including:

- Date and time of arrival at ED and of entry to study
- PRAM scores: hourly during stay in ED, then 4-hourly after admission
- Reasons for escalation of therapy
- Adverse effects of therapy
- Failure to tolerate HiFlo, or requirement for sedation to do so
- Date and time of starting and stopping IV bronchodilators and HiFlo
- Date and time of weaning off oxygen
- Date and time of meeting discharge criteria
- Date and time of discharge

The observation sheets will be incorporated into and harmonised with existing clinical documents in the 3 centres, and study setup will incorporate training and development of site-specific documents, to ensure ownership and acceptance by clinical staff. Anonymised data will be entered by trained research nurses electronically at each site onto an online password protected database (REDCap®) designed by the Brighton and Sussex CTU specifically for the study. A separate CRF pack will be provided to guide research nurses and ensure data consistency at the 3 centres.

Once the data have been cleaned and the database locked, the data will be transferred securely to the trial statistician for descriptive analysis by trial arm.

9.2 Data handling and record keeping

The data will be kept and handled according to the BSCTU data management plan.

9.3 Access to Data

Direct access will be granted to authorised representatives from the Sponsor, host institution and the regulatory authorities to permit trial-related monitoring, audits and inspections- in line with participant consent.

9.4 Archiving

- archiving will be authorised by the Sponsor following submission of the end of trial report
- all essential documents will be archived for a minimum of 5 years after completion of trial
- destruction of essential documents will require authorisation from the Sponsor

10 TRIAL MANAGEMENT, MONITORING, AUDIT & INSPECTION

The Brighton and Sussex Clinical Trials Unit (UKCRC Number 66) will take on the role of overseeing the management of the study. A trial manager (TM) will work closely with the research team to ensure that timelines are met, monitor and track recruitment and also undertake quality assurance monitoring visits to ensure the study is being conducted in accordance with the protocol. The TM will support the set up of the sites, ensuring all documentation and processes are in line with research governance and HRA processes.

Monthly trial management group (TMG) meetings with the CI, Data Manager (DM), statistician and PIs and research nurses from the sites will oversee the study progress. Recruitment and data updates will be discussed to highlight any issues and to ensure they can be resolved in a timely manner.

A DM will develop the case report form and oversee the quality of the data and will assist the statistician with ensuring the database is ready for analysis. An electronic data management system (REDCap®) will be used.

A trial steering committee (TSC) will consist of the TMG and the other co-applicants, together with 3 independent members (a lay member - parent of a child with asthma, a paediatrician with relevant expertise, and a statistician). The TSC will meet every 6 months, will receive reports from the TMG and will oversee the progress of the study. With its independent membership it will also review the data and safety issues, fulfilling the role for this feasibility trial of a data and safety monitoring board. Financial management for the study will be overseen by the trial manager with oversight for the project by Head of the Research Department at UHSussex (Scott Harfield).

11 ETHICAL AND REGULATORY CONSIDERATIONS

11.1 Research Ethics Committee (REC) review& reports

The protocol, informed consent form, patient’s legal representative/parent’s information sheet and any applicable documents will be submitted to the appropriate Research Ethics Committee (REC) and Health ResearchAuthority (HRA) for written approval according to applicable regulations. Approval by regulatory bodies of both the protocol and the consent form must be obtained before any participant is enrolled.

All substantial amendments to the original approved documents will be also sent to the appropriate REC and the HRA, for written approval according to applicable regulations; and will not be implemented until the a favourable opinion is granted. A determination will be made regarding whether a new consent needs to be obtained from participants who provided consent, using a previously approved consent form.

All correspondence with the REC will be retained in the Trial Master File/CI within 30 days of the anniversary date on which the favourable opinion was given, and annually until the trial is declared ended. The CI will notify the REC of the end of the trial, and also if the trial is ended prematurely, detailing the reasons for the premature termination. Within one year after the end of the trial, the CI will submit a final report with the results to the REC, including any publications/abstracts.

11.2 Peer review

The HiFlo ASA project proposal was successful in Competition 35 of the NIHR Research for Patient Benefit Programme, after two stages of independent peer review by the programme's designated expert advisory panel.

The study protocol for this feasibility study was further developed and discussed by researchers from the three participating centres, with involvement from the Brighton and Sussex CTU.

11.3 Patient and Public Involvement (PPI)

The following local patient groups have participated in the trial design and will also be informed of the findings of the study: - KSS Young People's Advisory Group (YPAG) - KSS Parent and Carer (PaC) Advisory Group

Patient and public involvement was sought at different stages in the development of this proposal, as follows:

- In January 2017 we contacted parents whose children had been admitted with acute asthma to get their feedback on research into novel methods of respiratory support in children with acute asthma. This initial feedback of their experiences encouraged us specifically to plan a study on high flow humidified oxygen in acute asthma in children.
- Subsequently, during the development of the Stage 1 RfPB application, we presented the project outline to the Kent Surrey and Sussex Young Persons' Advisory Group (KSS YPAG - part of the Generation R Alliance) and to the parallel Parents' group. These groups included children with asthma and their parents. Their detailed feedback was instrumental in eveloping the research strategy. Specifically:
    - o we were encouraged to pursue a delayed consent strategy - both children and parents understood the need for this and felt that in an emergency situation they would prefer this model for recruitment in a trial of HiFlo.
    - o they changed our thinking about how to ensure that families were aware that the trial was running in their emergency department. They did not feel that putting up posters (our planned strategy) was sufficient, and felt that all families who might conceivably be involved should be given a brief information leaflet at the time of booking in to the emergency department.
- Between the Stage 1 and Stage 2 submissions we have obtained further feedback as follows:
    - o We have again presented the project to the KSS YPAG and Parents' group. We organised parallel workshops of parents and young people aimed at improving the Plain English Summary (PES). Their excellent input resulted in a complete rewriting of the PES, and they made further comments on the revised version by email.
    - o We have made use of an RDS small grant to recruit a Lived Experience Advisory Panel (LEAP) – a group of 6 to 8 parents whose young children have been admitted with ASA, together with four children with experience of ASA. This group is providing disease-specific PPI into the study.

We will continue patient and public input, building on the above initiatives, as follows:

- KSS YPAG and Parents' group will continue to be involved in the development of participant information materials: the leaflet to be given to families at when they book in at the emergency department, and the subsequent detailed participant information sheet to be given at the time families are approached for deferred consent.
- The Lived Experience Advisory Panel (LEAP) - see above - with specific recent experience of acute asthma treatment will provide continuing input into the research. Their involvement has been costed in the proposal, and has been described in more detail in section 6.6 "Qualitative assessments". Specifically the LEAP will be involved in:
    - advising on participant information materials, in conjunction with the YPAG
    - designing the questions to be asked in the telephone interview
    - designing the short global satisfaction questionnaire to be given to parents and older children at hospital discharge
    - advising on recruitment during the study
    - interpreting the results of the study and co-designing the subsequent definitive RCT
    - defining the important outcomes for the definitive RCT

The LEAP will meet at six monthly intervals during the study to monitor and advise on recruitment (including problem solving any difficulties), support qualitative data analysis, and assist in the interpretation of study results. They will be given basic training in qualitative methods. At the point of dissemination, they will also be involved in planning activities.

11.4 Regulatory Compliance

Sponsor will ensure that this study is conducted in accordance with the protocol, the principles of the Declaration of Helsinki, Good Clinical Practice (GCP) and in full conformity with relevant regulations.

As mentioned in 11.1 above the trial will not commence until a Favourable REC opinion and subsequent HRA apprvoval is received.

Each participating site will provide their confirmation of capacity and capability to run the study prior to commencing recruiting.

11.5 Protocol compliance

Protocol non-compliances (departures from the approved protocol) will be recorded and reported to the trial manager, Chief Investigator and Sponsor, and subsequently to the TSC.

Deviations from the protocol which are found to frequently recur are not acceptable, will require immediate action and could potentially be classified as a serious breach.

11.6 Notification of Serious Breaches to GCP and/or the protocol

A "serious breach" is a breach which is likely to effect to a significant degree –

(a) the safety or physical or mental integrity of the participants of the trial; or
(b) the scientific value of the trial

The sponsor will be notified immediately of any case where the above definition applies during the trial conduct phase.

11.7 Data protection and patient confidentiality

All investigators and trial site staff must comply with the requirements of the General Data Protection Regulation 2018 with regards to the collection, storage, processing and disclosure of personal information and will uphold the Act's core principles. Details of this can be found here: https://www.hra.nhs.uk/about-us/news-updates/gdpr-guidance-researchers/

11.8 Financial and other competing interests for the chief investigator, PIs at each site and committee members for the overall trial management

No financial or other competing interests to declare.

11.9 Indemnity

In the case of any harm to participants arising from the management or design of the research, the NHS indemnity scheme will apply.

An agreement will be signed between UHSussex and Vapotherm covering insurance and indemnity in relation to the loaned equipment.

11.10 Amendments

Amendments which arise during the course of the study will be reviewed by the Sponsor prior to submission to the relevant authority, following the Sponsor's SOP.

11.11 Access to the final trial dataset

The final full dataset will be available to the CI, the site PIs, the study statistician and members of the steering group. Aspects of the dataset will be available to the other members of the study team.

12 DISSEMINATION POLICY

12.1 Dissemination policy

Because this is a feasibility trial, the important aspects of dissemination concern use of the trial data in designing a full RCT and preparing an application to fund this.

Dissemination will therefore be principally:

1. Among the study team

2. Among the PPI groups involved

3. Among stakeholders (both professional and patient/parent groups) to be involved in the full multicentre RCT.

It is intended to publish the protocol of this feasibility trial.

12.2 Authorship eligibility guidelines and any intended use of professional writers

Authorship on any publications resulting from this trial will be limited to those who have contributed directly to the trial - this will include the co-applicants on the grant application and other collaborators and members of the study team who have contributed.

13 REFERENCES

1. Eldridge SM, Chan CL, Campbell MJ, Bond CM, Hopewell S, Thabane L, et al. CONSORT 2010 statement: extension to randomised pilot and feasibility trials. BMJ. 2016;355:i5239. Epub 2016/10/26.
2. UK A. Asthma Facts and Statistics. 2018; Available from: https://www.asthma.org.uk/about/media/facts-and-statistics/.
3. Martinez FD, Vercelli D. Asthma. Lancet. 2013;382(9901):1360-72. Epub 2013/09/18.
4. Brand PL, Baraldi E, Bisgaard H, Boner AL, Castro-Rodriguez JA, Custovic A, et al. Definition, assessment and treatment of wheezing disorders in preschool children: an evidence-based approach. The European respiratory journal. 2008;32(4):1096-110. Epub 2008/10/02.
5. BTS/SIGN. British guideline on the management of asthma2016.
6. Physicians RCo. Why asthma still kills: the National Review of Asthma Deaths (NRAD) Confidential Enquiry report. London: RCP, 2014.
7. Papiris SA, Manali ED, Kolilekas L, Triantafillidou C, Tsangaris I. Acute severe asthma: new approaches to assessment and treatment. Drugs. 2009;69(17):2363-91. Epub 2009/11/17.
8. Haq I, Gopalakaje S, Fenton AC, McKean MC, C JOB, Brodlie M. The evidence for high flow nasal cannula devices in infants. Paediatric respiratory reviews. 2014;15(2):124-34. Epub 2014/01/30.
9. Mukherjee M, Stoddart A, Gupta RP, Nwaru BI, Farr A, Heaven M, et al. The epidemiology, healthcare and societal burden and costs of asthma in the UK and its member nations: analyses of standalone and linked national databases. BMC medicine. 2016;14(1):113. Epub 2016/08/30.
10. Chawla J, Seear M, Zhang T, Smith A, Carleton B. Fifty years of pediatric asthma in developed countries: how reliable are the basic data sources? Pediatr Pulmonol. 2012;47(3):211-9. Epub 2011/09/10.
11. Merrill C, Owens PL. Reasons for Being Admitted to the Hospital through the Emergency Department for Children and Adolescents, 2004: Statistical Brief #33. Healthcare Cost and Utilization Project (HCUP) Statistical Briefs [Internet]. Rockville (MD): Agency for Healthcare Research and Quality (US); 2006.
12. Bahadori K, Doyle-Waters MM, Marra C, Lynd L, Alasaly K, Swiston J, et al. Economic burden of asthma: a systematic review. BMC pulmonary medicine. 2009;9:24. Epub 2009/05/21.
13. Jones AJ, Mathew S, Wong SW, Patel M, Equi A, Sukhani S, et al. A regional audit of high-flow nasal cannula therapy use for bronchiolitis in London district general hospitals. Arch Dis Child. 2017;102(3):296-7. Epub 2017/01/26.
14. Beggs S, Wong ZH, Kaul S, Ogden KJ, Walters JA. High-flow nasal cannula therapy for infants with bronchiolitis. The Cochrane database of systematic reviews. 2014(1):CD009609. Epub 2014/01/21.
15. Yoder BA, Stoddard RA, Li M, King J, Dirnberger DR, Abbasi S. Heated, humidified high-flow nasal cannula versus nasal CPAP for respiratory support in neonates. Pediatrics. 2013;131(5):e1482-90. Epub 2013/04/24.
16. Locke RG, Wolfson MR, Shaffer TH, Rubenstein SD, Greenspan JS. Inadvertent administration of positive end-distending pressure during nasal cannula flow. Pediatrics. 1993;91(1):135-8. Epub 1993/01/01.
17. Dysart K, Miller TL, Wolfson MR, Shaffer TH. Research in high flow therapy: mechanisms of action. Respiratory medicine. 2009;103(10):1400-5. Epub 2009/05/27.
18. McKiernan C, Chua LC, Visintainer PF, Allen H. High flow nasal cannulae therapy in infants with bronchiolitis. The Journal of pediatrics. 2010;156(4):634-8. Epub 2009/12/29.
19. Schibler A, Pham TM, Dunster KR, Foster K, Barlow A, Gibbons K, et al. Reduced intubation rates for infants after introduction of high-flow nasal prong oxygen delivery. Intensive Care Med. 2011;37(5):847-52. Epub 2011/03/04.
20. Hilliard TN, Archer N, Laura H, Heraghty J, Cottis H, Mills K, et al. Pilot study of vapotherm oxygen delivery in moderately severe bronchiolitis. Arch Dis Child. 2012;97(2):182-3. Epub 2011/11/22.
21. Kepreotes E, Whitehead B, Attia J, Oldmeadow C, Collison A, Searles A, et al. High-flow warm humidified oxygen versus standard low-flow nasal cannula oxygen for moderate bronchiolitis (HFWHO RCT): an open, phase 4, randomised controlled trial. Lancet. 2017;389(10072):930-9. Epub 2017/02/06.
22. Franklin D, Babl FE, Schlapbach LJ, Oakley E, Craig S, Neutze J, et al. A Randomized Trial of High-Flow Oxygen Therapy in Infants with Bronchiolitis. The New England journal of medicine. 2018;378(12):1121-31. Epub 2018/03/22.
23. Arora B, Mahajan P, Zidan MA, Sethuraman U. Nasopharyngeal airway pressures in bronchiolitis patients treated with high-flow nasal cannula oxygen therapy. Pediatric emergency care. 2012;28(11):1179-84. Epub 2012/11/02.
24. Baudin F, Buisson A, Vanel B, Massenavette B, Pouyau R, Javouhey E. Nasal high flow in management of children with status asthmaticus: a retrospective observational study. Annals of intensive care. 2017;7(1):55. Epub 2017/05/24.
25. Ballestero Y, De Pedro J, Portillo N, Martinez-Mugica O, Arana-Arri E, Benito J. Pilot Clinical Trial of High-Flow Oxygen Therapy in Children with Asthma in the Emergency Service. The Journal of pediatrics. 2018;194:204-10 e3. Epub 2018/01/15.

26. Gilbert RE, Mok Q, Dwan K, Harron K, Moitt T, Millar M, et al. Impregnated central venous catheters for prevention of bloodstream infection in children (the CATCH trial): a randomised controlled trial. Lancet. 2016;387(10029):1732-42. Epub 2016/03/08.
27. Sim J, Lewis M. The size of a pilot study for a clinical trial should be calculated in relation to considerations of precision and efficiency. Journal of clinical epidemiology. 2012;65(3):301-8. Epub 2011/12/16.
28. Teare MD, Dimairo M, Shephard N, Hayman A, Whitehead A, Walters SJ. Sample size requirements to estimate key design parameters from external pilot randomised controlled trials: a simulation study. Trials. 2014;15:264. Epub 2014/07/06.
29. Lyttle MD, Gamble C, Messahel S, Hickey H, Iyer A, Woolfall K, et al. Emergency treatment with levetiracetam or phenytoin in status epilepticus in children-the EcLiPSE study: study protocol for a randomised controlled trial. Trials. 2017;18(1):283. Epub 2017/06/21.
30. Lyttle MD, Rainford NEA, Gamble C, Messahel S, Humphreys A, Hickey H, et al. Levetiracetam versus phenytoin for second-line treatment of paediatric convulsive status epilepticus (EcLiPSE): a multicentre, open-label, randomised trial. Lancet. 2019. Epub 2019/04/22.
31. Rydman RJ, Roberts RR, Albrecht GL, Zalenski RJ, McDermott M. Patient satisfaction with an emergency department asthma observation unit. Academic emergency medicine : official journal of the Society for Academic Emergency Medicine. 1999;6(3):178-83. Epub 1999/04/07.
32. Wong DL, Baker CM. Pain in children: comparison of assessment scales. Pediatric nursing. 1988;14(1):9-17. Epub 1988/01/01.
33. Bime C, Gerald JK, Wei CY, Holbrook JT, Teague WG, Wise RA, et al. Measurement characteristics of the childhood Asthma-Control Test and a shortened, child-only version. NPJ primary care respiratory medicine. 2016;26:16075. Epub 2016/10/21.
34. Chalut DS, Ducharme FM, Davis GM. The Preschool Respiratory Assessment Measure (PRAM): a responsive index of acute asthma severity. The Journal of pediatrics. 2000;137(6):762-8. Epub 2000/12/13.
35. Malterud K, Siersma VD, Guassora AD. Sample Size in Qualitative Interview Studies: Guided by Information Power. Qualitative health research. 2015. Epub 2015/11/29.
36. Burnard P. A method of analysing interview transcripts in qualitative research. Nurse education today. 1991;11(6):461-6. Epub 1991/12/01.
37. Hazell CM, Jones CJ, Hayward M, Bremner SA, O'Connor DB, Pinfold V, et al. Caring for Caregivers (C4C): study protocol for a pilot feasibility randomised control trial of Positive Written Disclosure for older adult caregivers of people with psychosis. Pilot and feasibility studies. 2017;3:63. Epub 2017/12/05.

14. APPENDICES

14.1 Appendix 1 – Schedule of Events

**Procedures**<br><br>**HiFlo Study Schedule of Events**	**VISIT**				
	**Screening phase (From ED attendance)**	**Enrolment (T0)**	**Treatment phase (T1, T2, … T:>24, TC)**	**Actual hospital discharge (TD)**	**Follow up (TF - 1-2 weeks post-discharge)**
Inform parents of the study (poster, leaflet)	X		X		
Eligibility assessment	X				
Demographics & medical history	X				
Observations: physical examination[1], vital signs[2], PRAM scoring[3], O_2 requirement[4]	X		X		
Eligibility check & Randomisation		X			
Early HiFlo / Standard Therapy (incl. treatment escalation & weaning)			X		
Deferred informed consent[5]			X		
Routine bloods (pCO_2)			X		
Concomitant medications			X		
Adverse event assessments			X		
CRF completion and data query resolution			X	X	X
End of study questionnaire				X	
Qualitative interviews with health professionals and parents					X

[1] Includes evaluation of suprasternal retraction, scalene muscle contraction, air entry, wheezing, work of breathing (respiratory distress), chest findings and cardiovascular status (CVS).

[2] Includes RR, HR, SaO_2, CRT, Temp

[3] Hourly in ED, 4-hourly thereafter

[4] Includes Flow, FiO_2

[5] 12-72 hours post enrolment

14.2 Appendix 2 – Amendment History

Amendment No.	Protocol version no.	Date issued	Author(s) of changes	Details of changes made
NSA007	4	10/08/2023	Paul Seddon<br>Hector Rojas	Change in the definition of recruitment in two sections of the protocol that describe primary feasibility objectives and outcome measures, for this deferred consent study. The changes are needed to clarify terminology and to comply with the NIHR definition of the term "recruitment".<br><br>The original definition of recruitment in the protocol was based on classical study design with consent prior to randomisation. However, operationally for this study, we used the term enrolment to describe those who were randomised, and recruitment = randomised + deferred consent obtained.
AM-04-S	3	27/05/2021	Hector Rojas	Change of Sponsor to University Hospitals Sussex NHS Foundation Trust (UHSussex)
AM-03-S	2	02/11/2020	Paul Seddon<br>Hector Rojas	Update of CONSORT diagram.<br><br>"Re-escalating inhaled bronchodilator therapy to hourly or more frequent" added to list of escalations in tables of section 6.3.2<br><br>Added descriptions for “verbal telephone consent” and the consent process for “bereaved parents” to section 6.4.1<br><br>Improved definition of "Time of Meeting discharge criteria (TC)" in section 6.5.1<br><br>Updated definition of Adverse Event (AE) in section 7.1<br><br>Added description of the intention to perform a per protocol analysis in addition to the primary intention to treat analysis in section 8.5<br><br>Some clarifications of ambiguous text, plus correction of typos and formatting
