# Supplementary material for: High-Flow Humidified Oxygen as an Early Intervention in Children With Acute Severe Asthma: Protocol for a Feasibility Randomized Controlled Trial
Source: JMIR Res Protoc. 2024 Mar 28;13:e54081. doi: 10.2196/54081 (PMC11009849; doi:10.2196/54081)
Supplement: Multimedia Appendix 2 [file resprot_v13i1e54081_app2.pdf]

National Institute for Health Research

Research for Patient Benefit
Review Summary

Research details

**Reference Number**	PB-PG-1217-20024
**Research Title**	High Flow humidified oxygen as an early intervention in children with Acute Severe Asthma. A feasibility study (HiFlo ASA).
**Chief Investigator**	Professor Paul Seddon
**Contracting Organisation**	Brighton and Sussex University Hospitals NHS Trust
**Total Amount Requested**	£246,404.00
**Reviewer Number**	1

Table Of Contents

1. Reviewer Expertise
2. Relevance of the proposed research
3. Quality of the proposed work
4. Strength of the research team
5. Impact of the proposed work
6. Value for money
7. Involvement of patients and the public
8. Additional Comments

1. Reviewer Expertise

**Please indicate the nature of your expertise by clicking on the appropriate tick box(es) below:**
Clinician in the same/a very similar field, Researcher in the same/a very similar field

If the tick boxes above do not adequately capture the nature of your expertise, please briefly provide details in the box below (or use it to give us more detail about your expertise if you wish):
I am a clinical academic with a research interest in the management of severe paediatric asthma.

2. Relevance of the proposed research

i) How relevant and important is the proposed research to the priorities and needs of the NHS, and does it offer a health/healthcare solution with demonstrable benefit to patients?<br>ii) Does the application demonstrate an awareness and understanding of previous relevant research or developments in this area?<br>iii) To what extent does the proposed research add distinct value or advance existing knowledge in this area, taking into account wider ongoing or completed research?
The research question of optimal management of a severe asthma attack in children is an important one. It is true that beyond initial burst therapy, we have little evidence for which intervention is best as a next step.<br>A sound summary of the current literature is provided. However, given the recently published pilot trial that the authors have acknowledged, I am uncertain of the added value here. The current study proposed to look at feasibility and utility of high flow nasal cannula oxygen therapy in children who have not responded within the first 4 hours of presentation to A&E, the published pilot trial has shown that high flow nasal cannula therapy was superior to conventional oxygen therapy in reducing respiratory distress, but had no impact on any other aspects of asthma management, including need for additional therapies. The published trial is a very similar size and has very similar criteria. I therefore am uncertain of the added benefit here.

3. Quality of the proposed work

**Research Design**<br>i) How appropriate is the research design in relation to the stated objectives?<br>ii) To what extent is the proposed design and methodology for all elements of the research well defined, appropriate, valid and feasible within the timeframe and resources requested?<br>iii) What are the strengths and weaknesses of the research design as proposed?<br>iv) To what extent does the research show originality and innovation?
There are several issues about the trial design that need justification:<br>1. Why has the age range 2-11 years been chosen? The objective is to assess efficacy of high flow nasal cannula oxygen in all children with an acute attack of wheeze/asthma, so why the cut-off of 11 years? Older children and adults may also respond to the intervention, I cannot see a justification for the age range.<br>2. I accept the authors have distinguished preschool wheezers from older children in their planned stratification, however, the inclusion criteria include burst therapy and oral corticosteroids. Many / most preschool children would not be prescribed oral corticosteroids for an acute wheeze episode, how will this be allowed for?<br>3. I think there may be maximal added value from this trial if the age is limited to preschool children. They are the most frequently admitted with acute wheezing, and they are the group for whom we have little evidence of any additional benefit from intravenous bronchodilators or steroids, and we know little about efficacy of delivery of inhaled bronchodilators in this age group. The pathophysiology of acute wheezing in preschool children is different to that in school-age children, with more contribution likely from infection in the preschool age group. The authors propose the advantage of using high flow nasal cannula oxygen is to help minimise secretions and provide humidified, warm oxygen, rather than dry oxygen. The preschool age group may benefit from this because of the age of the children, and that increased secretions likely contribute more to their wheeze, rather than smooth muscle contraction.<br>4. I am uncertain whether only children who require oxygen will be randomised and included. The inclusion criteria state failed response to initial burst therapy and PRAM score >/= 6, and that score would include low sats, but need for oxygen or sats alone are not part of the inclusion criteria. I am very unfamiliar with the PRAM score and have tried to look up how it is used, but cannot see whether low sats are a definite requirement for a score of 6 or greater. The use of the PRAM score and whether only those children who need oxygen after initial therapy will be included needs to be clarified. If children with normal sats are to be included, it is difficult to see what the advantage of the high flow nasal cannula oxygen would be. Although some CPAP is generated, I am uncertain how, given the physiology of acute asthma and tendency for air trapping, how CPAP would benefit, other than if it is used for oxygenation. I can see that high flow might be beneficial if oxygen therapy is needed, or prior to consideration of added ventilator support, such as NIV, but I cannot see how it would work if introduced before either of these requirements.<br>5. The BTS guidelines state children with sats <94% should receive oxygen, but the PRAM score of 6, as stated in the application includes sats between 90 and 92%. Should the trial not include the same sats cut-off as the BTS guidelines? Otherwise children will receive conventional oxygen therapy sooner and the benefit of high flow may not be determined?<br><br>The strengths include recruitment early in the emergency dept, with significant thought having been paid to consent procedures. But, I am concerned about the very basics of the trial design and how the benefit of high flow nasal cannula will be determined given the discrepancy between BTS guidelines and proposed PRAM score, and the lack of clarity about whether only hypoxic children will be included. The age range for inclusion also needs to be explained.

Has the research been designed with reference to an appropriate review of the existing literature?
Yes

**Work plan and proposed management arrangements**<br>i) How appropriate are the work plan and project management arrangements, and do they give confidence that proposed milestones will be met within the specified timeframe?<br>ii) Are the necessary clinical, academic or organisational links needed to support the research, in place?<br>iii) Please assess whether the key risks identified by the applicants have been adequately addressed, such as:<br><br>• Ethical, scientific, technical and organisational challenges<br>• Intellectual Property (IP) and commercial issues<br>iv) Please identify any additional barriers to the proposed work, not mentioned in the application form, that the applicants are likely to encounter in meeting their milestones<br>v) If this application has been submitted to the NIHR Programme Grants for Applied Research Programme, what

added value would be delivered over and above the dividends from the individual elements?
The work plan has been clearly laid out, and with the inclusion of a trial manager and clinical trials unit, it is likely that milestones will be met.<br>I am uncertain who will actually obtain the consent, in terms of hands-on work on the ground. I do not think any of the PIs will be taking consent, and I am uncertain how much research nurse time has been allowed for consent.<br>There is another discrepancy in the protocol compared to BTS guidelines - the protocol states ability to maintain sats >92% in air as a primary outcome measure and hospital discharge criteria, but BTS guidelines state sats >94% in air should be the cut-off.

**Plain English summary**<br>The plain English summary is intended for an interested audience, who are not necessarily specialists. The summary should be written at roughly the same level as an article in a newspaper. With this in mind, please comment on the following:<br><br>i) Does the plain English summary give a clear explanation of the research?<br>• Does it help you carry out your review? If not, why not?<br>• Is the language used appropriate and clear? If not, where are the problems?<br>• Are scientific terms, abbreviations and jargon explained? If not, which terms need explanation?<br>ii) If this research is funded, the plain English summary will be published on a variety of websites, without the rest of this application form. Could this plain English summary be used on its own to describe the proposed research? If not, what further information is needed?<br><br>Further information for researchers on how to write a plain English summary and what to include in a summary is available online at NIHR Make it clear www.involve.nihr.ac.uk/makeitclear.
The lay summary is clear except for the paragraph that describes the consent process. It states there will be a delay in asking for consent but families will be told the research is taking place. This is vague and does not explain that families will be entered into the trial initially and consent will be obtained later, and they can withdraw later if they want to.<br>The lay summary does not justify the age group chosen, it would be unclear to lay members of the public why only children aged between 2 and 11 are to be included.<br>The remainder of the lay summary is satisfactory.

4. Strength of the research team

i) How well are the roles of the team members described? Is the overall team well coordinated?<br>ii) On the basis of track record in relevant areas, how qualified are the applicants to undertake the work using the methodologies proposed?<br>iii) To what extent does the research team have the necessary breadth and depth of expertise to deliver the proposed work (*e.g.* as judged by publication output and previous research funding)?<br>iv) How could the strength of the research team be improved?<br>v) If the lead applicant is inexperienced, does he/she have appropriate support (*e.g.* from their organisation and/or more senior colleagues) to deliver the work plan?
There are PIs with expertise in interventional clinical trials in the acute setting. The CI does not have a track record of interventional trials, but the emergency dept colleagues have recruited to acute trials and therefore recruitment should be successful.<br>It is unclear why these 3 centres have been chosen.<br>The inclusion of a CTU also makes recruitment likely to be successful and achieved.

5. Impact of the proposed work

**Dissemination, outputs and anticipated impact**<br>NIHR aims to fund research that has the potential to be of significant benefit to the NHS, patients and the public. To support this, the applicants should consider how they will achieve impact from the outset. This helps them to identify potential impact and beneficiaries, and plan processes by which the research can directly, or incrementally over time, lead to change.<br><br>i) Have the applicants clearly expressed the problem and outlined how this research contributes towards a solution? Are the planned outputs appropriate and sufficient?<br>ii) Is it clear from the application who or which groups (including, as appropriate, healthcare planners, clinicians, patients and/or policy makers) are expected to benefit the most, how they will be engaged and communicated with, and if appropriate methods of engagement and dissemination are planned?<br>iii) Have the applicants set out appropriate activities and resources to achieve their impact goals? Is there a realistic trajectory and estimate of timescales for the benefit to reach patients/ public/ health and care services? Are there clear connections between outputs, engagement processes and impact goals?<br>iv) Have the applicants clearly considered what follow-on support they might need to generate or upscale impact, and how they might leverage further investment?<br>v) Have the applicants sufficiently and correctly identified any potential barriers they might face (e.g. IP, regulatory and acceptability) and have they properly considered how these may be overcome?<br>vi) Have the applicants clearly and realistically outlined the anticipated impacts, the likely scale of these impacts (both in the shorter and longer terms), and the sequence in which the impacts might occur?
Little is included about impact, output and dissemination as the applicants state this is a feasibility study to help plan a full RCT.<br>Dissemination focusses on publication of the trial protocol and findings.

6. Value for money

i) Taking into consideration the costs associated with undertaking the research, is there sufficient justification for the resources requested? Are all costs essential for the work proposed?<br>ii) Are appropriate resources set aside for patient and public involvement - including plans for a training and support budget?<br>iii) Where relevant, are resources to support impact - other than those for public and patient involvement - included and appropriate?<br>iv) Taking into account the total cost of the research, including the NHS costs, to what extent does the research provide value for money?<br>v) If required, are funds requested for NHS support and treatment costs appropriate and justified?
The majority of resources requested are for salary for all PIs and trial manager. The costs appear appropriate.<br>NHS costs are appropriate

7. Involvement of patients and the public

**Was there any patient and public involvement in the application?**	Yes

i) What is your assessment of the patient and public involvement (if any) in the development of the application, including involvement in: Identifying the research topic; prioritising the research questions; preparing the application (e.g. contributing to the research design); and identifying potential impact?<br>ii) What is your assessment of any proposed plans for patient and public involvement throughout the life of the research? Can you identify particular strengths, weaknesses and/or areas for improvement?

There has been extensive involvement and patients and public in the application design and the plan is to continue to obtain feedback from families who are in the trial as well.<br>The proposed plans for future PPI is very good.

8. Additional Comments

If you have any additional comments, not covered by the sections above, please provide them below.

National Institute for
Health Research

Research for Patient Benefit
Review Summary

Research details

**Reference Number**	PB-PG-1217-20024
**Research Title**	High Flow humidified oxygen as an early intervention in children with Acute Severe Asthma. A feasibility study (HiFlo ASA).
**Chief Investigator**	Professor Paul Seddon
**Contracting Organisation**	Brighton and Sussex University Hospitals NHS Trust
**Total Amount Requested**	£246,404.00
**Reviewer Number**	2

Table Of Contents

1. Reviewer Expertise
2. Relevance of the proposed research
3. Quality of the proposed work
4. Strength of the research team
5. Impact of the proposed work
6. Value for money
7. Involvement of patients and the public
8. Additional Comments

1. Reviewer Expertise

Please indicate the nature of your expertise by clicking on the appropriate tick box(es) below:
Clinician in the same/a very similar field, Researcher in the same/a very similar field

If the tick boxes above do not adequately capture the nature of your expertise, please briefly provide details in the box below (or use it to give us more detail about your expertise if you wish):
I am a clinical academic in Paediatric Respiratory Medicine based in the UK.

2. Relevance of the proposed research

i) How relevant and important is the proposed research to the priorities and needs of the NHS, and does it offer a health/healthcare solution with demonstrable benefit to patients?<br>ii) Does the application demonstrate an awareness and understanding of previous relevant research or developments in this area?<br>iii) To what extent does the proposed research add distinct value or advance existing knowledge in this area, taking into account wider ongoing or completed research?
i) The use of high-flow nasal cannula oxygen therapy (HFNC) has been rapidly adopted in quite a varied range of patients with respiratory failure from neonatology to adult high dependency/critical care. Perceived benefits include ease of use and the fact that HFNC is generally well tolerated by babies and children. The results of several large scale RCTs in preterm neonates and infants with bronchiolitis are now becoming available - with mixed results in terms of actual efficacy. In paediatric practice HFNC is now often used for a range of respiratory problems (including acute wheeze) without good quality evidence to support its use. Another important angle I think here is that HFNC can be used and set up in a ward-based setting rather than in critical care and it is important that it is not regarded as a replacement for HDU or critical care (HFNC devices can deliver surprisingly high amounts of positive pressure and up to 100% oxygen - leaving little reserve for further decompensation depending on the settings used).<br>Investigating the use of HFNC in children with acute severe asthma is an important topic as I think it is creeping in to practice for this indication, makes some but not complete physiological sense, and both efficacy and safety need to be studied.<br><br>ii) Yes, relatively little work performed in this specific area.

iii) This is obviously a preliminary study that will inform future larger studies, to this end I believe it will signifcantly add to current knowledge.

3. Quality of the proposed work

**Research Design**<br>i) How appropriate is the research design in relation to the stated objectives?<br>ii) To what extent is the proposed design and methodology for all elements of the research well defined, appropriate, valid and feasible within the timeframe and resources requested?<br>iii) What are the strengths and weaknesses of the research design as proposed?<br>iv) To what extent does the research show originality and innovation?
i) The study design is appropriate to investigate feasibility with an allied qualitative thread to the work to investigate parental and health care staff's opinions and experiences.<br><br>ii) There is a good level of detail provided in the application. It is an admirably ambitious approach involving deferred consent and rapid commencement of HFNC in an ED setting - note that ethical approval has not been obtained yet and this may not be straightforward and could be time consuming and/or involve some redesigning of the approach. Resources, etc seem appropriate.<br><br>iii) A major strength is the 'real-life' pragmatic approach that is proposed - will certainly gather a good deal of authentic feasibility-type information. A weakness to the methodology to my mind is that the treatment other than HFNC does not seem to be 'protocolised' in either arm i.e. use of which IV bronchodilators and when at what dose. How will nebulised bronchodilators be given with HFNC prongs in? Is there a maximum FiO2 allowed in the HFNC arm?<br><br>iv) The application is original and ambitious in my opinion.

Has the research been designed with reference to an appropriate review of the existing literature?
Yes

**Work plan and proposed management arrangements**<br>i) How appropriate are the work plan and project management arrangements, and do they give confidence that proposed milestones will be met within the specified timeframe?<br>ii) Are the necessary clinical, academic or organisational links needed to support the research, in place?<br>iii) Please assess whether the key risks identified by the applicants have been adequately addressed, such as:<br><br>• Ethical, scientific, technical and organisational challenges<br>• Intellectual Property (IP) and commercial issues<br>iv) Please identify any additional barriers to the proposed work, not mentioned in the application form, that the applicants are likely to encounter in meeting their milestones<br>v) If this application has been submitted to the NIHR Programme Grants for Applied Research Programme, what added value would be delivered over and above the dividends from the individual elements?
i) Detailed project management plan is proposed with checkpoints and regular progress checks - I have no concerns in this regard.<br><br>ii) Yes<br><br>iii) Ethical permission obviously needs to be obtained, deferred consent may need discussion in this regard. It goes without saying that the study must be executed in an as clinically safe way as possible - patients must be observed closely with care escalated as necessary, this is likely to involve transfer to ITU and possible intubation so this has to be robust and delivered in the clinical settings to be used in the study with an appropriate level of observation.

iv) None that are not mentioned - but if obtaining ethical permission is not straightforward this could take time and/or a redesign of the study; other obvious potential is problems with recruitment, deferred consent, etc

v) Not applicable I think

Plain English summary
The plain English summary is intended for an interested audience, who are not necessarily specialists. The summary should be written at roughly the same level as an article in a newspaper. With this in mind, please comment on the following:

i) Does the plain English summary give a clear explanation of the research?

- Does it help you carry out your review? If not, why not?
- Is the language used appropriate and clear? If not, where are the problems?
- Are scientific terms, abbreviations and jargon explained? If not, which terms need explanation?

ii) If this research is funded, the plain English summary will be published on a variety of websites, without the rest of this application form. Could this plain English summary be used on its own to describe the proposed research? If not, what further information is needed?

Further information for researchers on how to write a plain English summary and what to include in a summary is available online at NIHR Make it clear www.involve.nihr.ac.uk/makeitclear.

i) The plain English summary is easy for me to follow but uses acronyms and some jargon, e.g. ASA seem unnecessary

ii) As above, needs to be looked at again, deferred consent sentences don't really explain the gist of that process in my opinion.

4. Strength of the research team

i) How well are the roles of the team members described? Is the overall team well coordinated?
ii) On the basis of track record in relevant areas, how qualified are the applicants to undertake the work using the methodologies proposed?
iii) To what extent does the research team have the necessary breadth and depth of expertise to deliver the proposed work (*e.g.* as judged by publication output and previous research funding)?
iv) How could the strength of the research team be improved?
v) If the lead applicant is inexperienced, does he/she have appropriate support (*e.g.* from their organisation and/or more senior colleagues) to deliver the work plan?

i) Well-described and clear roles delineated

ii) Combined track record is strong

iii) Appropriate range of expertise and multidisciplinarity

iv) No obvious need for any changes

v) Experienced PI

5. Impact of the proposed work

Dissemination, outputs and anticipated impact
NIHR aims to fund research that has the potential to be of significant benefit to the NHS, patients and the public.

To support this, the applicants should consider how they will achieve impact from the outset. This helps them to identify potential impact and beneficiaries, and plan processes by which the research can directly, or incrementally over time, lead to change.<br><br>i) Have the applicants clearly expressed the problem and outlined how this research contributes towards a solution? Are the planned outputs appropriate and sufficient?<br>ii) Is it clear from the application who or which groups (including, as appropriate, healthcare planners, clinicians, patients and/or policy makers) are expected to benefit the most, how they will be engaged and communicated with, and if appropriate methods of engagement and dissemination are planned?<br>iii) Have the applicants set out appropriate activities and resources to achieve their impact goals? Is there a realistic trajectory and estimate of timescales for the benefit to reach patients/ public/ health and care services? Are there clear connections between outputs, engagement processes and impact goals?<br>iv) Have the applicants clearly considered what follow-on support they might need to generate or upscale impact, and how they might leverage further investment?<br>v) Have the applicants sufficiently and correctly identified any potential barriers they might face (e.g. IP, regulatory and acceptability) and have they properly considered how these may be overcome?<br>vi) Have the applicants clearly and realistically outlined the anticipated impacts, the likely scale of these impacts (both in the shorter and longer terms), and the sequence in which the impacts might occur?
i) Yes in my opinion<br><br>ii) Largely the researchers are aiming to generate feasibility data to inform a further larger study - they will feedback to members of PPI groups involved in the study design<br><br>iii) These are limted (see answer to ii)<br><br>iv) As above, does not seem directly relevant to this application<br><br>v) Yes<br><br>vi) Little other than informing future study designs and applications

6. Value for money

i) Taking into consideration the costs associated with undertaking the research, is there sufficient justification for the resources requested? Are all costs essential for the work proposed?<br>ii) Are appropriate resources set aside for patient and public involvement - including plans for a training and support budget?<br>iii) Where relevant, are resources to support impact - other than those for public and patient involvement - included and appropriate?<br>iv) Taking into account the total cost of the research, including the NHS costs, to what extent does the research provide value for money?<br>v) If required, are funds requested for NHS support and treatment costs appropriate and justified?
i) An ambitious project to deliver - costs seem appropriate<br><br>ii) Costs budgeted for meetings appropriately<br><br>iii) Seem to be limited to publication of protocol<br><br>iv) Yes to a reasonable extent<br><br>v) Yes

7. Involvement of patients and the public

**Was there any patient and public involvement in the application?**	Yes

i) What is your assessment of the patient and public involvement (if any) in the development of the application, including involvement in: Identifying the research topic; prioritising the research questions; preparing the application (e.g. contributing to the research design); and identifying potential impact?<br>ii) What is your assessment of any proposed plans for patient and public involvement throughout the life of the research? Can you identify particular strengths, weaknesses and/or areas for improvement?
I) There is evidence of good involvement of the local YPAG and subsequent adaptation of the proposal based on feedback received<br><br>ii)) Can also see a clear plan for ongoing PPI

8. Additional Comments

If you have any additional comments, not covered by the sections above, please provide them below.

National Institute for Health Research

Research for Patient Benefit
Review Summary

Research details

**Reference Number**	PB-PG-1217-20024
**Research Title**	High Flow humidified oxygen as an early intervention in children with Acute Severe Asthma. A feasibility study (HiFlo ASA).
**Chief Investigator**	Professor Paul Seddon
**Contracting Organisation**	Brighton and Sussex University Hospitals NHS Trust
**Total Amount Requested**	£246,404.00
**Reviewer Number**	3

Table Of Contents

1. Reviewer Expertise

2. Relevance of the proposed research

3. Quality of the proposed work

4. Strength of the research team

5. Impact of the proposed work

6. Value for money

7. Involvement of patients and the public

8. Additional Comments

1. Reviewer Expertise

**Please indicate the nature of your expertise by clicking on the appropriate tick box(es) below:**
Clinician in the same/a very similar field

If the tick boxes above do not adequately capture the nature of your expertise, please briefly provide details in the box below (or use it to give us more detail about your expertise if you wish):

2. Relevance of the proposed research

i) How relevant and important is the proposed research to the priorities and needs of the NHS, and does it offer a health/healthcare solution with demonstrable benefit to patients?<br>ii) Does the application demonstrate an awareness and understanding of previous relevant research or developments in this area?<br>iii) To what extent does the proposed research add distinct value or advance existing knowledge in this area, taking into account wider ongoing or completed research?
i) this research proposal addresses a common and significant health risk for children in the UK. Asthma affects children of all ages beyond infancy and is associated with chronic morbidity as well as acute severe and life threatening illness requiring high intensity care and resource demands. There is a potential for this study to lead to demonstrable benefit to patients if the results show less deterioration and more rapid recovery from acute episodes of asthma or other illnesses causing respiratory difficulty with wheezing.<br>ii) Previous relevant research is clearly identified and considered in the application<br>iii)The proposed research aims to expand on the results of existing trials that left unanswered questions in relation to the timing of application of Hi Flow therapy and to enrol appropriate numbers of subjects to give statistically significant data that will show the feasibility of funding a further randomised controlled trial of this intervention as a treatment option to be more widely used

3. Quality of the proposed work

**Research Design**<br>i) How appropriate is the research design in relation to the stated objectives?<br>ii) To what extent is the proposed design and methodology for all elements of the research well defined, appropriate, valid and feasible within the timeframe and resources requested?<br>iii) What are the strengths and weaknesses of the research design as proposed?<br>iv) To what extent does the research show originality and innovation?
I) The research design seems appropriate for some of the stated objectives in that it will indicate if application of HiFlow for wheezy children at time of arrival in the Emergency Department is realistic and practical. It also emphasises the testing of use of deferred consent to research in a study of acutely ill children in the ED and has extensive detail on the involvement of service users and young people with lived experience of the condition in the information to be shared with patients and carers and in the interpretation of results. The inclusion of behavioural psychology and follow up interviews for qualitative research in this area is valuable<br>ii) The strengths are<br>a) the multi-professional approach to this research question, the inclusion of Emergency Medicine is a study of a problem that presents to that department and where initial therapy is critical to outcomes.<br>b) the involvement of service users from the start ad throughout the research with active participation of young people and response options from young children in relation to the effect and acceptability of treatment<br>c) inclusion of training and motivational support for staff in all areas involved in the study to aim for maximum recruitment and implementation of the study protocol<br>d) it may be both a strength and a weakness that the inclusion criteria are so wide as this will allow study of children and young people with a range of causes of wheeze and may indicate the need for separation into more specific diagnostic groups for further analysis of response to treatments<br>The weaknesses appear to be:<br>a) lack of diagnostic detail on the cause of wheeze - the protocol defines all children over age 2 presenting with wheeze that fails to respond to 3 doses of inhaled bronchodilator as severe asthma and potential study subjects. There may be different reason for wheeze that may result in differing responses to the application of HiFlow - in particular the associated CPAP effect with its potential risk for air leak. As the authors describe not all the children who present with wheeze will have asthma - there is no mention of the possibility of e.g. foreign body as a cause of wheezing and although humidified oxygen might be appropriate first line treatment for this presentation till definitive intervention the application of high flow and positive pressure is not.<br>b) There is mention of the need for sedation to allow application of the Hi Flow equipment and such treatment may itself compromise respiratory drive and would generally be avoided in an ED setting<br>c) the outcome as time till discharge home and the statement that the child will remain in ED till recovery seems incomplete when admission to a Children's Ward or PICU may be clinically indicated although this may depend on the Children's emergency facilities in the individual hospitals and so could be acceptable if acknowledged by the research teams.<br>d) There is no mention of the treatment the patient may have received prior to arrival at the ED but the use of dry oxygen in an ambulance could affect the response to treatment commenced in the ED, likewise use of neulised bronchodilator either in Primary Care of in an ambulance could alter the impact of high humidity oxygen on arrical<br>e) There is little mention of the agreed level of initial therapy prior to allocation as severe asthma. If following SIGN/BTS guidelines an anticholinergic bronchodilator would be considered as initial treatment in Hospital but the research protocol appears to assume use of a beta agonist only with no mention of how that is being administered - again whether by nebuliser or MDI and spacer with or without oxygen. The inclusion of an anticholinergic may affect the viscosity of respiratory secretions with a potential to influence the impact of enhanced humidification and may be a further variable that should be considered in the analysis of results<br>f) The protocol states that the HiFlow will start at 1l/min but this is not a high flow and may result in the study being more on the effect of adequately humidified gas rather than or in parallel to the effect of high flow - in this regard the use of humidification for the control group should be more clearly defined.<br>iv) The research does not aim to be original and recognises its building on previous studies to try to justify a larger research study and as such it is innovative in its approach to recruitment and deferred consent to facilitate early intervention in an acute clinical situation

Has the research been designed with reference to an appropriate review of the existing literature?
Yes

**Work plan and proposed management arrangements**<br>i) How appropriate are the work plan and project management arrangements, and do they give confidence that proposed milestones will be met within the specified timeframe?

ii) Are the necessary clinical, academic or organisational links needed to support the research, in place?
iii) Please assess whether the key risks identified by the applicants have been adequately addressed, such as:

- Ethical, scientific, technical and organisational challenges
- Intellectual Property (IP) and commercial issues

iv) Please identify any additional barriers to the proposed work, not mentioned in the application form, that the applicants are likely to encounter in meeting their milestones
v) If this application has been submitted to the NIHR Programme Grants for Applied Research Programme, what added value would be delivered over and above the dividends from the individual elements?

I) Work plan and project management appear appropriate. The milestones can be met but might be adversely affected during the winter bronchiolitis season when every children's Emergency facility is seeing many young infants with wheeze and prioritisation of research recruitment and availability of equipment could be compromised
ii) Ethical challenges relate to the question of deferred consent and are addressed by the applicants although the appropriateness of this approach may only be evident as the study progresses and with the views of the LEAP and lay panel over time. It appears the researchers have experience of use of this approach and so are alert to its risks as well as its benefits. It is not clear what is the time scale for seeking consent form the child's parents or if the child or young person themselves will also be asked for their consent. This may be particularly relevant if there is an early adverse event associated with use of the new therapy such as acute air leak that may be coincidental or consequent to its use.
Scientific challenges appear to have been addressed, it would be important to include what organisational and technical contingency plans are in place for servicing or supporting sue of the equipment in and out of hours and if there is a backup option in the event of equipment failure.
There is little detail of IP and commercial issue management particularly in relation to supply of free equipment from a commercial company
iv) Potential barriers may be the need to retrain staff when there are changes of junior medical or nursing staff on each of the research sites as well as the difficulty in avoiding word of mouth spread of opinion on the benefits or otherwise of any new equipment or therapy introduced to a general hospital site both among staff and the public.v) The applicants identify but do not emphasise the risk of over enthusiastic introduction of untested or evidence free new techniques that may be at high cost to the health service. This research could provide further data on the relevance of humidification of inspired gases in treatment of respiratory conditions and allergic disorders that could have very wide relevance in paediatric health care

Plain English summary
The plain English summary is intended for an interested audience, who are not necessarily specialists. The summary should be written at roughly the same level as an article in a newspaper. With this in mind, please comment on the following:

i) Does the plain English summary give a clear explanation of the research?

- Does it help you carry out your review? If not, why not?
- Is the language used appropriate and clear? If not, where are the problems?
- Are scientific terms, abbreviations and jargon explained? If not, which terms need explanation?

ii) If this research is funded, the plain English summary will be published on a variety of websites, without the rest of this application form. Could this plain English summary be used on its own to describe the proposed research? If not, what further information is needed?

Further information for researchers on how to write a plain English summary and what to include in a summary is available online at NIHR Make it clear www.involve.nihr.ac.uk/makeitclear.

I) yes the plain English summary is clear and helpful. The language is appropriate though it is likely to be difficult for young patients to understand the implications of potential risks.
There is no indication of how the information is being translated or tested with patients and parents who do not speak English or have literacy difficulties.
The only abbreviation that may need explanation relates to the asthma severity score and quality of life scoring. the plain English summary is not adequate for publication without some explanation that not all the subjects in the study have a diagnosis of asthma.

4. Strength of the research team

i) How well are the roles of the team members described? Is the overall team well coordinated?<br>ii) On the basis of track record in relevant areas, how qualified are the applicants to undertake the work using the methodologies proposed?<br>iii) To what extent does the research team have the necessary breadth and depth of expertise to deliver the proposed work (*e.g.* as judged by publication output and previous research funding)?<br>iv) How could the strength of the research team be improved?<br>v) If the lead applicant is inexperienced, does he/she have appropriate support (*e.g.* from their organisation and/or more senior colleagues) to deliver the work plan?
I) very well coordinated<br>ii) well qualified<br>iii) this appears appropriate for the research that is described<br>iv) Possibly with representation from children's nursing in emergency or PICU care<br>v) N/A

5. Impact of the proposed work

**Dissemination, outputs and anticipated impact**<br>NIHR aims to fund research that has the potential to be of significant benefit to the NHS, patients and the public. To support this, the applicants should consider how they will achieve impact from the outset. This helps them to identify potential impact and beneficiaries, and plan processes by which the research can directly, or incrementally over time, lead to change.<br><br>i) Have the applicants clearly expressed the problem and outlined how this research contributes towards a solution? Are the planned outputs appropriate and sufficient?<br>ii) Is it clear from the application who or which groups (including, as appropriate, healthcare planners, clinicians, patients and/or policy makers) are expected to benefit the most, how they will be engaged and communicated with, and if appropriate methods of engagement and dissemination are planned?<br>iii) Have the applicants set out appropriate activities and resources to achieve their impact goals? Is there a realistic trajectory and estimate of timescales for the benefit to reach patients/ public/ health and care services? Are there clear connections between outputs, engagement processes and impact goals?<br>iv) Have the applicants clearly considered what follow-on support they might need to generate or upscale impact, and how they might leverage further investment?<br>v) Have the applicants sufficiently and correctly identified any potential barriers they might face (e.g. IP, regulatory and acceptability) and have they properly considered how these may be overcome?<br>vi) Have the applicants clearly and realistically outlined the anticipated impacts, the likely scale of these impacts (both in the shorter and longer terms), and the sequence in which the impacts might occur?
I) The problem is clearly stated and the research is relevant to identifying potential for improvement at an early stage and also the risk of innovation creep if this intervention has limited use in the clinical setting described. The outcomes are appropriate, there could be additional benefit in separating the humidification question from the high flow/CPAP element in the analysis of results<br>ii) yes , communication is well described but this is a feasibility study proposal so dissemination fo results is likely to be limited to the need for further trials<br>iii) Yes the results of this study will not be at a stage to disseminate to the public and this is recognised by the applicants<br>iv) as a feasibility study there is very little detail about any follow up beyond the qualitative research interviews.<br>v) as a feasibility study these questions are of limited relevance but appear to have been considered in accepting involvement of a commercial company as supplier of equipment<br>vi) there is limited detail of impact assessment beyond the anticipated need for a larger randomised controlled trail as a result of the research

6. Value for money

i) Taking into consideration the costs associated with undertaking the research, is there sufficient justification for the resources requested? Are all costs essential for the work proposed?<br>ii) Are appropriate resources set aside for patient and public involvement - including plans for a training and support budget?<br>iii) Where relevant, are resources to support impact - other than those for public and patient involvement - included and appropriate?<br>iv) Taking into account the total cost of the research, including the NHS costs, to what extent does the research provide value for money?<br>v) If required, are funds requested for NHS support and treatment costs appropriate and justified?
I) resources appear to be justified apart from costs of courier that is not described in the application<br>ii) yes there are appropriate resources specified for patient and public involvement and these appear to have been identified by the public involvement advisory group<br>iii) this does not appear to apply to this application<br>iv) if all aims are achieved with consideration of the additional information that could be extracted on respiratory humidification effects, this research appears to be value for money<br>v) NHS funds are justified

7. Involvement of patients and the public

**Was there any patient and public involvement in the application?**	Yes

i) What is your assessment of the patient and public involvement (if any) in the development of the application, including involvement in: Identifying the research topic; prioritising the research questions; preparing the application (e.g. contributing to the research design); and identifying potential impact?<br>ii) What is your assessment of any proposed plans for patient and public involvement throughout the life of the research? Can you identify particular strengths, weaknesses and/or areas for improvement?
I) there has been impressive effort to involve patients and public and on-going involvement with evidence that advice from the initial application has been followed .<br>ii) there is clear intention to continue to involve and adapt to patient and public opinion during the study using existing and newly formed groups and taking advantage of the psychology expertise of one of the research team

8. Additional Comments

If you have any additional comments, not covered by the sections above, please provide them below.
The study involves use of one type of HiFlow equipment and it is possible that differing technology may affect the effective CPAP and level of humidification delivered to the airways. The researchers should acknowledge this and it would be helpful to know then how their findings might apply to other applications.

National Institute for Health Research

Research for Patient Benefit
Review Summary

Research details

**Reference Number**	PB-PG-1217-20024
**Research Title**	High Flow humidified oxygen as an early intervention in children with Acute Severe Asthma. A feasibility study (HiFlo ASA).
**Chief Investigator**	Professor Paul Seddon
**Contracting Organisation**	Brighton and Sussex University Hospitals NHS Trust
**Total Amount Requested**	£246,404.00
**Reviewer Number**	4

Table Of Contents

1. Reviewer Expertise
2. Relevance of the proposed research
3. Quality of the proposed work
4. Strength of the research team
5. Impact of the proposed work
6. Value for money
7. Involvement of patients and the public
8. Additional Comments

1. Reviewer Expertise

**Please indicate the nature of your expertise by clicking on the appropriate tickbox(es) below:**
Patient or service user with direct experience of this area

If the tick boxes above do not adequately capture the nature of your expertise, please briefly provide details in the box below (or use it to give us more detail about your expertise if you wish):

2. Relevance of the proposed research

i) Is there a clear and credible reason for doing this research? If there is, what is it?
ii) Is this research important or relevant to patients or carers? Why is that?
iii) Could the results of the research make a difference to patients or carers? If yes, how would they make a difference? If not, why not?

Asthma can be incredibly scary and frightening for the patient, family members and people that view asthma attacks. For that reason alone if there is something that can lessen the impact of the effects of asthma acute attack then it is worth trying. The scientific abstract indicates that in certain cases the 1st and 2nd line treatments have limited effectiveness and significant side effects. Its also increases the number of hospital attendance and admissions for children which I am sure has other effects on their families and the larger community, such as mental anguish, time off work, use of hospital beds, NHS costs etc. In my opinion if there is something we can try that will reduce the consequences of an acute asthma attack, we should try it. This is about the feasibility of doing a RCT.

For the above reasons the research is important but we have to be clear that in other respiratory conditions it has shown some promise.My understanding is for the purpose of this research, it would become like a 2nd line treatment once burst therapy has failed. I am not sure what is meant by some promise, has it shown more or less effectiveness than standard 2nd line treatment previously.

Why were the 3 sites chosen,is it because they have higher attendance rates for ASA or another reason?

The main results to be looked at are a) time readiness for hospital discharge. Getting out of hospital with an excellent discharge plan clearly communicated is the best mentally, physically and financially for patients, families,

NHS and the wider community. How are the patients GP's engaged, is it through the process of a discharge letter or is there another mechanism.

The other main result is b) treatment failure requiring escalation of therapy. What is actually measured there is it the cost, physical and mental impact on patients, carers, time out of school and other support services?
Trails do have wider impacts and consequences for children and carers time off school, time off work for parents. Has that been taken in to consideration?

In writing and asking all of the above I think its a good idea to try it.

3. Quality of the proposed work

Research Design
i) Are the outcomes the researchers are planning to measure appropriate? Will the research ultimately benefit patients, service users, carers and/or the public? Why is that the case? Are there other outcomes that are more important? If so, what are they?
ii) Have the researchers taken a realistic approach to recruiting people to participate in their research? Could this be improved and if so, how? Do you think people are likely to agree to take part? Would you be willing to take part in the research or suggest to a friend that they did?

In plain English the outcomes to me seem to be a) how long it takes to get the patients to be discharged and b) how many fail that require additional treatment. They seem to measure success and failure. I don't know what the questionnaires after are like but I would hope it would measure the mental and physical impact for not just the NHS but the patients and cares. Having a sick child often requires time off work, having to pay additional child care costs, effects on people careers etc
Ultimately this research will measure if the use of HIFlo is worth using as a standard pathways after carrying out an RCT so it to early to say whether this will have a positive effect on patients, carers the public but it will give the NHS some guidance of whether this is a pathway that needs further research.
From a adult patient point of view the most important outcome is getting the patient home safe and sound with minimal impact on normal life. Reduction in costs for the NHS is great and very important but is a secondary outcome. Going to and staying in hospital has a hugh impact on life, again I am unsure if this is measured in questionairres as a postive consequence or as a soft outcome. I think these outcomes should be taken into consideration.
It seems a small sample size of 70 children,I am assuming they are known users of ED and /or have suffered ASA and use the services of the institutes involved. I am assuming this is also standard sample size for these types of trails. I could not say whether it could be improved on. Patient recruitment looks like it is from Jul/Aug 2019 till about April 21 but the follow up looks like it ends in May/June 21 for the last cohorts, is that normal in these type of trails?
In an emergency generally we panic, if they know about the trails before hand i.e contacting known families so there is an awareness before the emergency. People may ask for 2nd opinions in ED which can effect cost and time. Is the local CCG's and GP's aware? Is that necessary?
Personally I would prefer HiFLo it seems a more natural way but I hate the thought of tubes in my nostrils so I am not sure of what experience the children identified will have of this and if that will have an impact on the professionals treating them at the time, will it be awkward for the children, is sedation involved?
The team know there are some some success with HiFLo in other in a) babies and b) ASA later when rescue therapy has failed. How big the success is for b) I am unsure.

Has the research team taken account of previous research in this area?	Yes

Work plan and proposed management arrangements
i) How are any plans for patient and public involvement in the research also referred to in the work plan and in the proposed management arrangements? Could the plan and the management arrangements be improved from this

perspective?
From section 5 ML there are 3 emergency medicines responsible for coordinating patient identification and recruitment and a Psychologist involved in capturing the experience of patients and caregivers. I am not sure how that manifests operationally. On average how many ASA patients do the sites have, is it more in one site than another, are the resources in the right place?<br>On Page 13 it talk about patients/cares being invited to participate in a structured telephone interview, so does that mean it is non mandatory, they do not have to participate f they do not want? . I know there is a satisfaction questionnaire given to parent after leaving the hospital but they can often end up in bin. Will the sampling group be large enough to learn from?. It talks about at least 10 patients been recruited at each site and getting feedback from 6-8 which means their is an anticipation of a 60% engagement in in feedback at a minimum. That quite high, is that normal for these type of projects?<br>I know that patients and parents as well as advisory group have been involved in the issue around deferred consent and advising on posters/leaflets to engage parents once they come in to ED. That is great. Operationally how are staff trained to engage/raise awareness with parents as both clinical and non clinical staff need to be involved. Its not just clinical staff that need to be engaged, awareness needs to be in the whole team. How will they get this research piece high on the agenda with all the other conflicting priorities. Can they use a volunteer/ambassador type role from some of the panel groups to raise awareness. It would be good to see samples of some of the proposed material they intend to produce for participant engagement. I am assuming that all patients and public involvement has taken into consideration different ethic groups and communication needs. There is evidence that the KSS YPAG and parents groups will be involved all the way through which is great - can any of them be used as ambassadors to support the trial operationally by attending staff meetings to keep it high on the agenda and sharing positive feedback and by keeping awareness alive in ED. Possible use of the LEAP group.<br>The percentage of time and cost for staff members involved, is that average? Are there provisions in place if staff change roles/move on and would that have a risk especially with patient involvement.<br>In getting feedback from children over the age of 4, would that be done face to face and how would that be managed?

**Plain English summary**<br>The plain English summary is intended for an interested audience, who are not necessarily specialists. The summary should be written at roughly the same level as an article in a newspaper. With this in mind, please comment on the following:<br><br>i) Does the plain English summary give a clear explanation of the research?<br>• Does it help you carry out your review? If not, why not?<br>• Is the language used appropriate and clear? If not, where are the problems?<br>• Are scientific terms, abbreviations and jargon explained? If not, which terms need explanation?<br>ii) If this research is funded, the plain English summary will be published on a variety of websites, without the rest of this application form. Could this plain English summary be used on its own to describe the proposed research? If not, what further information is needed?<br><br>Further information for researchers on how to write a plain English summary and what to include in a summary is available online at NIHR Make it clear www.involve.nihr.ac.uk/makeitclear.
I thought it was good, plain English. It gave me an idea of the proposal and why it was proposed. I thought the language was suitable for someone like me .<br><br>When we read on of the most commonest reasons - what does not measure at? We know that its around 11%, why numbers not included.<br><br>I thought some of the wording like "current rescue therapy is unsatisfactory". Is that right surely it works in some cases but not all, it would have been good for me to see a percentage/number there.<br><br>Also "We think it works by", as HiFLo is already used in cases not only ASA, could the wording be different like we know from working with ASA at later stages it works by ....<br><br>How much money could it save the NHS?

Reference: PB-PG-1217-20024

It might also be wise to include something about the effects ASA and current treatment have on families i.e average length of stay in hospital, missing schoool etc

I don't think the public are afraid of numbers. We are used of it -

4. Strength of the research team

i) Does the research team appear to have the right mix of skills to carry out this research? For example, if the research involves looking at what nurses do, is there a nurse on the team? If not, how could the team be strengthened?
ii) Is there one or more suitably experienced member of the research team with responsibility for coordinating, supporting and delivering patient and public involvement activities? If not, how could this be addressed?
iii) Are patients, service users or carers included in the research team? And if so, is it clear what their role or roles will be and what they will bring to the research team?

The Chief Investigator has led on the PPI and 10% of their time is allocated to their involvement. Is that enough time to manage it? Is that similar to the average for these projects?. Who is responsible if the leads are not present.

The Principal Investigator is responsible to carry out training, is their provision included for when staff change and they may need to carry out additional training sessions.The members of the teams responsible for patient identification and recruitment have 7% of their time allocated. Is that sufficient. Who is responsible when they are not on shift and suitable patients are present.
From what I read all the team seems to be highly qualified and have relevant experience in trials so that is great
There will be Trail manager role and monthly meetings which will involve members from the sites.
6% time is allocated for the Psychologist involved. I am assuming pathways are in place for participants who present with additional needs i.e mental health, safeguarding etc

I am sure it is already considered by the people involved but often participants and families need the human touch and may need a listening ear at time and that needs to be accounted for .

5. Impact of the proposed work

Dissemination, outputs and anticipated impact
NIHR aims to fund research that has the potential to be of significant benefit to the NHS, patients and the public. To support this, the applicants should consider how they will achieve impact from the outset. This helps them to identify potential benefits and beneficiaries beyond the academic community, and plan processes by which the research can directly or incrementally over time, lead to change in the ‘real world’.

i) Have the applicants clearly expressed a real-world problem and how their research contributes towards a ‘solution’? How well do the planned outputs match this aim? If not, what changes are needed?
ii) Have the applicants made it clear what impacts they are aiming to achieve from the research? Are these plans appropriate? Are they achievable? Do they seem realistic in terms of scale and timing? If not, what needs changing?
iii) Have the applicants clearly stated who will benefit from this research (e.g., patients, carers, clinicians, policy makers, healthcare planners) and how they will benefit? Are plans to engage and communicate with these individuals/groups appropriate? If not, what is missing?
iv) Have the applicants chosen suitable activities to achieve impact? Have they made it clear how the outputs, beneficiaries and planned impacts are linked? If not, what needs changing?

v) Are the applicants clear on what would be needed (e.g. more funding, further partnerships) to sustain or increase impact after the project? If not, what else needs to be considered?<br>vi) Have the applicants sufficiently considered the barriers they may face in achieving impact (e.g. regulations, intellectual property/rights, acceptability to users)? Have they adequately considered how to overcome these? If not, what is missing?
It is fairly clear that the feasibility study is to see if HiFlo will work as a pathways for ASA and to reduce hospitals admissions and stays as there is a problem. If so the next step would be an RCT. There is currently not a lot of evidence to say the current 2nd line treatment is effective. So the idea behind this feasibility study is to investigate the effectiveness using HiFlo and whether to conduct a RCT . Currently it is being used for some conditions but there is no convincing data and it could just be used without evidence and so an additional cost amounting in the NHS. This feasibility should be able to evidence base the effectiveness for an RCT. Its says this feel this type of therapy could be delivered without discomfort or adverse effects to the airways.<br>I did originally think the sample size was small but this is just to see whether there could be enough evidence to carry out an RCT. I thinks the plans and timings are fine.<br>I don't agree with deferred consent. The groups they have spoken to may be used of been in an emergency situation but I would not be happy for my child to receive treatment without me being fully aware of what was happening even in an emergency situation. At that stage 1st line intervention would have failed so there is time to talk to the parent. I am not really clear on the side effects of HiFlo, would all patients needs to be sedated?, increased chest infections, headaches? as a parent I would want to know more about this.<br>Staff would need to be trained on the set up and the change of settings and cleaning of on the machine. There is a turn over of staff and use of Locums in the NHS, I suspect training might need to be ongoing and not just the preparatory stage?<br>It is clear what the benefits of the research are and the links areas but from a human point of view it is not clear what the down sides are. It is also clear that this would be a feasibility study before deciding on a full RCT.<br>It is not clear what additional learning there is from this for example will all the patients identified have good asthma plans in place and after an ASA irrespective of which treatment is given, will there be a review of their current asthma plans and will they be signed posted to asthma charities for additional support, I think this could be included in some of the secondary outcomes.<br>I would not be happy with deferred consent and that may be need further exploration. The paper mentions the treatment as being an add-on but I would want to know more about the negative side-effects. There is also little evidence other than mentioning the booking in team, leaflets and poster how they would use non - clinical staff to support the study.

6. Value for money

The NIHR provides guidance on what can and cannot be included in the costs of research. CCF carries out an initial financial scrutiny of all applications received. A more detailed scrutiny of finances is always carried out on any application that is recommended for funding. For example, see NIHR Finance Form FAQs.<br>As a public reviewer, you are not expected to assess whether the entire research budget is costed correctly. However, comments on the following aspects are welcome:<br>i) Overall, does the research budget seem a reasonable investment of public money? Could it save health and social care costs in the long term?<br>ii) Are the resources set aside for patient and public involvement appropriate for the proposed activities? E.g. for training and support, travel and other expenses, staff salaries? For more see: INVOLVE's Involvement Cost Calculator. If not, how could it be improved?
Yes I think the research is valuable but I would want to know more about the treatment itself.<br>Continuous training should be looked at due to change over/movement of staff, I know Vapotherm will provide some initial training in the use of the portable machines where needed but ongoing training costs should be looked at.<br>What happens if the machines break and are not functioning who covers the costs of that?<br>Has the provider of the machines being asked to fund the feasibility study or is that against NHS rules? If so the proposed funding could probably fund 5-6 senior nurses positions<br><br>In saying that if the end outcome is a better treatment for the patient, shorter time in hospital, less cost for the NHS,

then yes its value for money

7. Involvement of patients and the public

**Was there any patient and public involvement in the application?**	Yes

i) What is your assessment of the patient and public involvement in the development of the application including involvement in: identifying the research topic; prioritising the research questions; preparing the application (*e.g.* contributing to the research design); and identifying potential impact?<br>ii) What is your assessment of any proposed plans for patient and public involvement throughout the life of the research? Can you identify particular strengths, weaknesses and/or areas for improvement?
I am not sure what makes up the parents and young people groups and how reflective they are of society but yes they have been involved. In the TSC group a lay member will be involved and they will meet every 6 months and oversee the progress. Not sure what criterion is in place to ensure that lay member is supported as working with clinical teams can be very over powering. They carried out an email survey with parents of children with asthma, presented the study to a parents/young people group. Discussed the issue of deferred consent and how to make people attending ED aware of the feasibility study. They will also be involved in the design of leaflet given to people and the information given about deferred consent. LEAP are involved through designing questionnaires, satisfactions questionnaire, defining important outcome for the RCT advising on recruitment and they will meet every 6 months.There is evidence of involvement but the quality of the involvement and how it is acted on, I cant comment. That would only be fully ascertained by asking some of the lay members involved.

8. Additional Comments

If you have any additional comments, not covered by the sections above, please provide them below.
none

National Institute for Health Research

Research for Patient Benefit Review Summary

Research details

**Reference Number**	PB-PG-1217-20024
**Research Title**	High Flow humidified oxygen as an early intervention in children with Acute Severe Asthma. A feasibility study (HiFlo ASA).
**Chief Investigator**	Professor Paul Seddon
**Contracting Organisation**	Brighton and Sussex University Hospitals NHS Trust
**Total Amount Requested**	£246,404.00
**Reviewer Number**	5

Table Of Contents

1. Reviewer Expertise
2. Relevance of the proposed research
3. Quality of the proposed work
4. Strength of the research team
5. Impact of the proposed work
6. Value for money
7. Involvement of patients and the public
8. Additional Comments

1. Reviewer Expertise

**Please indicate the nature of your expertise by clicking on the appropriate tick box(es) below:**
Clinician in a broadly related field

If the tick boxes above do not adequately capture the nature of your expertise, please briefly provide details in the box below (or use it to give us more detail about your expertise if you wish):

2. Relevance of the proposed research

i) How relevant and important is the proposed research to the priorities and needs of the NHS, and does it offer a health/healthcare solution with demonstrable benefit to patients?<br>ii) Does the application demonstrate an awareness and understanding of previous relevant research or developments in this area?<br>iii) To what extent does the proposed research add distinct value or advance existing knowledge in this area, taking into account wider ongoing or completed research?
1.Acute asthma/wheezing is a common presentation to paediatric units and A/E. Some children respond poorly to initial treatments and develop increasing respiratory failure. Potentially they require IV treatments, high dependency and may also need intubation and ventilation on PICU. High flow is well established to provide respiratory support in a wide variety of respiratory conditions. This is a study looking at the potential of early intervention with high flow in this group. This sinot a treatment that should be used routinely in all asthmatics as this would be unnecessary in most cases and routine use would be expensive. However in selected cases where initial responses to treatment is poor it may actually be cost effective particularly if it avoids high dependency or intensive care and leads to early discharge.It is also likely to be more comfortable for children and well tolerated.<br><br>2. There is good awareness of the current literature with 2 publications of the use of high flow in acute asthma but not in a randomised control trial.<br><br>3.This would advance knowledge on the management of acute asthma which is a common presentation. This is a preliminary study which potentially will lead onto a larger multicentre trial.

3. Quality of the proposed work

**Research Design**<br>i) How appropriate is the research design in relation to the stated objectives?<br>ii) To what extent is the proposed design and methodology for all elements of the research well defined, appropriate, valid and feasible within the timeframe and resources requested?<br>iii) What are the strengths and weaknesses of the research design as proposed?<br>iv) To what extent does the research show originality and innovation?
I have some comments in relation to the application of the high flow-- why start on 1L which is not really high flow, why not start on 5 or 6 l.? also going up to a maximum of 40l seems a bit high particularly for a smaller child. Should there be a lower high limit<br><br>1 the research design is appropriate to delivering the objectives.<br><br>2.see my comments above. Methodology is well defined,valid,appropriate and feasible within the time frame and resources particularly with involvement of 3 large centres. delayed consent seems appropriate.<br><br>strengths-- adequate patient numbers should be available, high flow technology well established, Weakness- it is obviously not possible to blind this study which may introduce some bias. However the asthma severity scores are very objective and should reduce this possibility.<br><br>not particularly innovative as this is an established and available treatment in other conditions associated with respiratory distress.But the work is original and asks a specific research question and is important as previously stated.

Has the research been designed with reference to an appropriate review of the existing literature?
Yes

**Work plan and proposed management arrangements**<br>i) How appropriate are the work plan and project management arrangements, and do they give confidence that proposed milestones will be met within the specified timeframe?<br>ii) Are the necessary clinical, academic or organisational links needed to support the research, in place?<br>iii) Please assess whether the key risks identified by the applicants have been adequately addressed, such as:<br>• Ethical, scientific, technical and organisational challenges<br>• Intellectual Property (IP) and commercial issues<br>iv) Please identify any additional barriers to the proposed work, not mentioned in the application form, that the applicants are likely to encounter in meeting their milestones<br>v) If this application has been submitted to the NIHR Programme Grants for Applied Research Programme, what added value would be delivered over and above the dividends from the individual elements?
1. work plan, project management and timeframes are satisfactory. The numbers should be achieved in the specified timeframe given that there are 3 centres and this is a common presentation.<br><br>2. yes excellent links with academic doctors in different specialties and a statistician.<br><br>3.There are no significant ethical, scientific, technical or organisdational issues with this study.<br><br>4. no additional barriers.<br><br>5. ? not applicable

**Plain English summary**

The plain English summary is intended for an interested audience, who are not necessarily specialists. The summary should be written at roughly the same level as an article in a newspaper. With this in mind, please comment on the following:<br><br>i) Does the plain English summary give a clear explanation of the research?<br>• Does it help you carry out your review? If not, why not?<br>• Is the language used appropriate and clear? If not, where are the problems?<br>• Are scientific terms, abbreviations and jargon explained? If not, which terms need explanation?<br>ii) If this research is funded, the plain English summary will be published on a variety of websites, without the rest of this application form. Could this plain English summary be used on its own to describe the proposed research? If not, what further information is needed?<br><br>Further information for researchers on how to write a plain English summary and what to include in a summary is available online at NIHR Make it clear www.involve.nihr.ac.uk/makeitclear.
The plain English summary gives a very clear explanation of the research. I did not need it to help me carry out my review as the rest of the application is well written and clear. There is no use of jargon and it is appropriate and clear. It could be used on its own to describe the research.

4. Strength of the research team

i) How well are the roles of the team members described? Is the overall team well coordinated?<br>ii) On the basis of track record in relevant areas, how qualified are the applicants to undertake the work using the methodologies proposed?<br>iii) To what extent does the research team have the necessary breadth and depth of expertise to deliver the proposed work (*e.g.* as judged by publication output and previous research funding)?<br>iv) How could the strength of the research team be improved?<br>v) If the lead applicant is inexperienced, does he/she have appropriate support (*e.g.* from their organisation and/or more senior colleagues) to deliver the work plan?
There is a very good mix in the team with academics as well as good representation from each of the 3 units and the different specialties- respiratory paediatrics, Intensive care and Accident and Emergency medicine. A statistician and psychologist complement the team.The group is well co-ordinated. A number of co applicants have strong academic credentials. The team should be able to delivery this research study. The team does not need to be strengthened. the lead applicant is extremely experienced.

5. Impact of the proposed work

**Dissemination, outputs and anticipated impact**<br>NIHR aims to fund research that has the potential to be of significant benefit to the NHS, patients and the public. To support this, the applicants should consider how they will achieve impact from the outset. This helps them to identify potential impact and beneficiaries, and plan processes by which the research can directly, or incrementally over time, lead to change.<br><br>i) Have the applicants clearly expressed the problem and outlined how this research contributes towards a solution? Are the planned outputs appropriate and sufficient?<br>ii) Is it clear from the application who or which groups (including, as appropriate, healthcare planners, clinicians, patients and/or policy makers) are expected to benefit the most, how they will be engaged and communicated with, and if appropriate methods of engagement and dissemination are planned?<br>iii) Have the applicants set out appropriate activities and resources to achieve their impact goals? Is there a realistic trajectory and estimate of timescales for the benefit to reach patients/ public/ health and care services? Are there clear connections between outputs, engagement processes and impact goals?<br>iv) Have the applicants clearly considered what follow-on support they might need to generate or upscale impact,

and how they might leverage further investment?<br>v) Have the applicants sufficiently and correctly identified any potential barriers they might face (e.g. IP, regulatory and acceptability) and have they properly considered how these may be overcome?<br>vi) Have the applicants clearly and realistically outlined the anticipated impacts, the likely scale of these impacts (both in the shorter and longer terms), and the sequence in which the impacts might occur?
This is essentially a pilot study which would lead on to a larger trial.<br><br>1.The problem has been clearly outlined and this research will contribute to an important and common condition presenting acutely. The potential impact of this high flow intervention is improved patient comfort, less time in hospital and less high dependency and intensive care. This would have a positive financial impact. The planned outputs are appropriate and sufficient.<br><br>2. patients would benefit significantly potentially avoiding unpleasant interventions and there would be cost savings.Disseminating the results is planned through meetings and engaging with public support groups.<br><br>3. I think that the resources are adequate to achieve the goals of the study. The trajectory is realisitic.There are close correlations between outputs,engagement processes and impact goals.<br><br>4. the applicants have considered the needs for a larger trial beyond this study which should attract funding.<br><br>5. there should be few barriers to achieving this study and these have been considered.<br><br>6. The impact of this study will be to design a definitive and larger multicentre trial.

6. Value for money

i) Taking into consideration the costs associated with undertaking the research, is there sufficient justification for the resources requested? Are all costs essential for the work proposed?<br>ii) Are appropriate resources set aside for patient and public involvement - including plans for a training and support budget?<br>iii) Where relevant, are resources to support impact - other than those for public and patient involvement - included and appropriate?<br>iv) Taking into account the total cost of the research, including the NHS costs, to what extent does the research provide value for money?<br>v) If required, are funds requested for NHS support and treatment costs appropriate and justified?
I think the costs are justified and have been carefully calibrated.<br><br>appropriate resources have been set aside for patient/public involvement.<br><br>I think that this research does provide value for money as the potential for role out across the UK could prove to be very cost effective.<br><br>all funds requested are justified.

7. Involvement of patients and the public

**Was there any patient and public involvement in the application?**	Yes

i) What is your assessment of the patient and public involvement (if any) in the development of the application, including involvement in: Identifying the research topic; prioritising the research questions; preparing the application (e.g. contributing to the research design); and identifying potential impact?

ii) What is your assessment of any proposed plans for patient and public involvement throughout the life of the research? Can you identify particular strengths, weaknesses and/or areas for improvement?
good involvement with support groups and use of psychologist to consider quality of life issues is very good<br><br>no improvement required

8. Additional Comments

If you have any additional comments, not covered by the sections above, please provide them below.
nil
